# Differentially Expressed Plasma MicroRNAs and the Potential Regulatory Function of Let-7b in Chronic Thromboembolic Pulmonary Hypertension

**DOI:** 10.1371/journal.pone.0101055

**Published:** 2014-06-30

**Authors:** Lijuan Guo, Yuanhua Yang, Jie Liu, Lei Wang, Jifeng Li, Ying Wang, Yan Liu, Song Gu, Huili Gan, Jun Cai, Jason X.-J. Yuan, Jun Wang, Chen Wang

**Affiliations:** 1 Beijing Key Laboratory of Respiratory and Pulmonary Circulation Disorders, Beijing Chao-Yang Hospital, Capital Medical University, Beijing, P.R. China; 2 Beijing Institute of Respiratory Medicine, Capital Medical University, Beijing, P.R. China; 3 Department of Physiology, Capital Medical University, Beijing, P.R. China; 4 Department of Cardiology, Beijing Chao-Yang Hospital, Capital Medical University, Beijing, P.R. China; 5 Department of Cardiac Surgery, Beijing Anzhen Hospital, Capital Medical University, Beijing, P.R. China; 6 Beijing Institute of Heart, Lung and Vessel Disease, Capital Medical University, Beijing, P.R. China; 7 Department of Medicine, University of Illinois at Chicago, Chicago, Illinois, United States of America; 8 Department of Beijing Hospital, Ministry of Health, Beijing, P.R. China; Medical University of South Carolina, United States of America

## Abstract

Chronic thromboembolic pulmonary hypertension (CTEPH) is a progressive disease characterized by misguided thrombolysis and remodeling of pulmonary arteries. MicroRNAs are small non-coding RNAs involved in multiple cell processes and functions. During CTEPH, circulating microRNA profile endued with characteristics of diseased cells could be identified as a biomarker, and might help in recognition of pathogenesis. Thus, in this study, we compared the differentially expressed microRNAs in plasma of CTEPH patients and healthy controls and investigated their potential functions. Microarray was used to identify microRNA expression profile and qRT-PCR for validation. The targets of differentially expressed microRNAs were identified *in silico*, and the Gene Ontology database and Kyoto Encyclopedia of Genes and Genomes pathway database were used for functional investigation of target gene profile. Targets of let-7b were validated by fluorescence reporter assay. Protein expression of target genes was determined by ELISA or western blotting. Cell migration was evaluated by wound healing assay. The results showed that 1) thirty five microRNAs were differentially expressed in CTEPH patients, among which, a signature of 17 microRNAs, which was shown to be related to the disease pathogenesis by *in silico* analysis, gave diagnostic efficacy of both sensitivity and specificity >0.9. 2) Let-7b, one of the down-regulated anti-oncogenic microRNAs in the signature, was validated to decrease to about 0.25 fold in CTEPH patients. 3) ET-1 and TGFBR1 were direct targets of let-7b. Altering let-7b level influenced ET-1 and TGFBR1 expression in pulmonary arterial endothelial cells (PAECs) as well as the migration of PAECs and pulmonary arterial smooth muscle cells (PASMCs). These results suggested that CTEPH patients had aberrant microRNA signature which might provide some clue for pathogenesis study and biomarker screening. Reduced let-7b might be involved in the pathogenesis of CTEPH by affecting ET-1 expression and the function of PAECs and PASMCs.

## Introduction

Chronic thromboembolic pulmonary hypertension (CTEPH) is characterized by continuously increased pulmonary vascular resistance due to unresolved emboli in major pulmonary arterials and/or pulmonary microvascular remodeling [1]–[3]. Recent epidemiology studies showed that the incidence of CTEPH in acute pulmonary thromboembolism survivors was about 2.7%–8.8% [4]–[6], and 2-year survival in untreated patients with a mean pulmonary artery pressure greater than 50 mmHg was as low as 10% [7]. However, recognition before CTEPH progression is difficult for the insidious onset and lack of effective biomarker of it.

MicroRNAs (miRNAs) are small endogenous non-coding RNAs that suppress gene expression post-transcriptionally by binding to the “seed sequences” in 3′ untranslated regions (UTRs) of target mRNAs. Dysregulation of miRNAs has been found in different diseases and biological processes [8]. Recent studies have shown that miRNAs were involved in pulmonary vascular remodeling and susceptibility of CTEPH [9], [10], as well as pulmonary arterial smooth muscle cells (PASMCs) malproliferation of pulmonary arterial hypertension (PAH) [11]–[12].

Circulating miRNAs, known as stable cell-free miRNAs in serum or plasma, are passively and selectively released to blood by various cells, and may act as transmitter or messenger in cell communication [13], [14]. During disease, aberrantly expressed miRNAs in the diseased cells are released into the circulation, and the circulating miRNA profile is endued with the disease properties [15]. Recently, circulating miRNAs have been extensively studied as potential blood-based biomarkers for disease diagnosis, especially in malignancies and cardiovascular diseases. Plasma miR-134 has been shown to be a specific biomarker for acute pulmonary thromboembolism [16].

Multiple pathophysiologic processes have been reported to contribute to the progression of CTEPH [17]–[19], including imbalance of endothelin-1 (ET-1), nitric oxide and prostacyclin, dysfunction of pulmonary arterial endothelial cells (PAECs), and malproliferation of PASMCs. ET-1 is a key vasoconstrictor especially in pulmonary circulation, and can cause proliferation of many cells involved in vascular remodeling. ET-1 level was elevated in CTEPH patients [18], and endothelin receptor antagonists (ETAs) have been applied for CTEPH treatment [20]. Transforming growth factor (TGF)-β plays important regulatory roles in the balance of cell proliferation and apoptosis. The abnormal activation of TGF-β/transforming growth factor beta receptor 1 (TGFBR1) signaling was involved in development of idiopathic PAH [21], [22]. Clarify the relationship between candidate miRNAs and these known mechanisms would intensify the recognition of disease pathogenesis.

Taking the complex pathophysiology of CTEPH and the extensive regulatory function of miRNAs into account, we hypothesized that circulating miRNA profile might reflect the miRNAs involved in the pathogenesis of CTEPH more comprehensively, thus could be used as candidate biomarker and shed light on the recognition of CTEPH pathogenesis. In this study, we identified a 17 miRNA signature in CTEPH plasma and investigated the potential functions of this signature *in silico*. Let-7b, one of the key miRNAs in it, was shown to influence ET-1 level and migration of PAECs and PASMCs.

## Materials and Methods

### Subjects

The study procedure was approved by the Medical Ethics Committee of Beijing Chao-Yang Hospital affiliated to Capital Medical University, and the written informed consent was obtained from each subject. The microarray cohort was composed of 10 CTEPH patients and 10 healthy control subjects. For further validation, another 40 pairs of subjects participated. The patients were recruited over approximate 4 years (from March, 2007 to November, 2010). Diagnosis of CTEPH was established based on standard protocol previously described [23]. Most of the CTEPH patients received the right heart catheterization. The exclusion criteria included malignancies, other cardiovascular diseases, and patients who had ever received any treatment for pulmonary hypertension except for anticoagulation.

### Plasma Collection and RNA Extraction

Whole blood (2 mL) was drawn into Vacutainer (anti-coagulated with EDTA, BD, New Jersey, US), and plasma isolation was done within 2 hours by centrifugation at 1000 g for 10 minutes. Total RNA of plasma was harvested with the TRI Reagent (Sigma Aldrich, St. Louis, US). For real time PCR, an internal control of 5 fmol synthetic Syn-cel-miR-39 (Qiagen, Valencia, CA) was added to plasma after mixing with TRI Reagent, as previously described [24].

### MiRNA Microarray and *In Silico* Analysis

The miRCURY LNA Array (version 14.0, Exiqon, Vedbaek, DK) was performed in microarray cohort. The dataset of microarray has been submitted to the repository of Gene Expression Omnibus, and the accession number is GSE56914. Random variance model (RVM) *t*-test was applied to filter the differentially expressed miRNAs. A greedy search, heuristic algorithm for making locally optimal choices at each stage with the hope of finding a global optimum, was taken to find different marker combinations, and during cross-validation of diagonal samples for each combination, seven diagnostic methods were applied [25]. More details can be found in File S1. TargetScan and miRDB were combined for target prediction. The Gene Ontology (GO) Database [26] and the KEGG PATHWAY Database [27] were used for functional investigation of target gene profile of differentially expressed miRNAs. The pathway analysis of let-7b alone was done using DIANA-miRPath [28].

### Real-Time Quantitative Reverse-Transcription Polymerase Chain Reaction

A stem-loop real-time quantitative reverse transcription (qRT) polymerase chain reaction (PCR) was used to validate the data obtained by microarray in an enlarged independent cohort. TaqMan MicroRNA Assay, TaqMan MicroRNA Reverse Transcription Kit, and TaqMan Gene Expression Master Mix (Applied Biosystems, Foster, US) were used. The real-time PCR was performed on the ABI PRISM7500 system (Applied Biosystems, Foster, US).

### Fluorescent Reporter Assay

The native 3′-UTR fragments of ET-1 and TGFBR1 containing the predicted hsa-let-7b binding sites were amplified from the human lung fibroblast cDNA of a health donor, and subcloned into pcDNA3.1/enhanced green fluorescent protein (EGFP) construct by *Not*I and *Xba*I (NEB, Ipswich, US) subsequently. The control constructs were generated by point mutation in the predicted “seed sequence” of hsa-let-7b using TaKaRa MutanBEST Kit (TaKaRa, Dalian, CHN). The primer sets were shown in Table S1 in File S1.

### Western Blotting and Enzyme-Linked Immunosorbent Assay

Expression of target genes TGFBR1 and ET-1 was detected by Western blotting and enzyme-linked immunosorbent assay (ELISA) respectively.

### Wound Healing Assay

Migration of human PAECs and PASMCs (ScienCell, California, US) was evaluated by wound healing assay [29].

### Statistical Analysis

Demographic and clinical characteristics of the subjects were described as mean ± SD, or median for abnormal distribution data. The Mann-Whitney *U* test, two-sample Kolmogorov-Smirnov test and 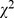 test were used to examine differences between two groups. Multiple comparison was done by two-way ANOVA with Student-Newman-Keuls method for Post Hoc Tests. Receiver-operating characteristic (ROC) curve analysis was also performed for candidate miRNA levels against CTEPH. Univariable linear and logistics regression analyses were conducted to evaluate the relationships between candidate miRNAs and clinically related indices. Age and sex were considered as controlled variables. SPSS 16.0 was used for all statistical analyses and *P*<0.05 (two-tailed) was considered statistically significant.

Additional details are available in File S1.

## Results

### Clinical Characteristics of Subjects

Ten CTEPH patients and 10 healthy controls matched in age and sex were involved in microarray cohort, and another 40 pairs in validation cohort. Baseline and clinical characteristics are shown in Table 1. In microarray cohort, all CTEPH patients received right heart catheterization, and acute vascular reaction test was positive in 1 out of 9 subjects (11.1%). The mean pulmonary artery pressure was 54.6 mmHg, and the pulmonary vascular resistance was 1071.2 dyn·s·cm^−5^. In validation cohort, 36 CTEPH patients received right heart catheterization, and acute vascular reaction was positive in 6 subjects (16.7%). The mean pulmonary artery pressure was 51.5 mmHg, and pulmonary vascular resistance was 1079.4 dyn·s·cm^−5^. There was no difference between CTEPH patients of the microarray and validation cohort in hemodynamic index, deep venous thrombosis, WHO functional class, 6-minute walk distance, acute vascular reaction and N-terminal pro-brain natriuretic peptide except C-reactive protein (Table S2 in File S1).

**Table 1:** Clinical characteristics of the subjects.

Characteristics	miRNA microarray		qRT-PCR	
	Healthy control (n = 10)	CTEPH patient (n = 10)	Healthy control (n = 40)	CTEPH patient (n = 40)
Age (yr)*	49.7 (10.1)	49.8 (10.1)	49.6 (9.5)	51.9 (11.6)
**Sex (Male/Total)**	6/10	6/10	24/40	25/40
**DVT (Positive/Total)**	-	6/10	-	12/30
MBP (mmHg)*		91.2 (4.4)		93.3 (12.0)
sPAP (mmHg)*†	-	86.3 (13.5)	-	89.5 (19.7)
mPAP (mmHg)*†	-	54.6 (13.3)	-	51.5 (12.0)
PVR (dyn•s•cm^−5^)*†	-	1071.2 (243.9)	-	1079.4 (598.1)
CI (L•min^−1^•m^−2^)*†	-	2.0 (0.6)	-	2.0 (0.6)
**WHO functional class**				
**I**	-	1/10	-	2/40
**II**	-	2/10	-	19/40
**III**	-	6/10	-	16/40
**IV**	-	1/10	-	3/40
6MWD (m)*‡	-	374.6 (27.0)	-	355.5 (109.0)
**AVR (Positive/Total)**	-	1/9	-	6/36
NTproBNP (pg/mL)*§	-	1436.6 (1577.1)	-	1456.2 (1433.5)
CRP (mg/L)*∥	-	6.0 (6.0)	-	4.7 (4.2)

Definition of abbreviations: DVT, deep venous thrombosis; MBP, mean blood pressure; sPAP, systolic pulmonary arterial pressure; mPAP, mean pulmonary arterial pressure; PVR, pulmonary vascular resistance; CI, cardiac index; 6MWD, 6-minute walk distance; AVR, acute vascular reaction; NTproBNP, N-terminal pro-brain natriuretic peptide; CRP, C-reactive protein. * Mean (SD); for microarray cohort, † n = 10, ‡ n = 7, § n = 8, ∥ n = 8; and for validation (qRT-PCR) cohort, † n = 36, ‡ n = 31, § n = 34, ∥ n = 29.

### Aberrantly Expressed Circulating MiRNAs in CTEPH Patients

To determine the differential circulating miRNA profile of CTEPH, we employed the miRCURY LNA Array (version 14.0) system with plasma extracted RNA of 10 CTEPH patients and 10 healthy controls. As displayed in Table S3 in File S1, the circulating miRNA profiles differed significantly between CTEPH patients and healthy controls. Of 1700 probes on this array, we found 15 up-regulated and 20 down-regulated miRBase included miRNAs in CTEPH patients compared with healthy controls at *P*<0.05.

**Figure 1:**
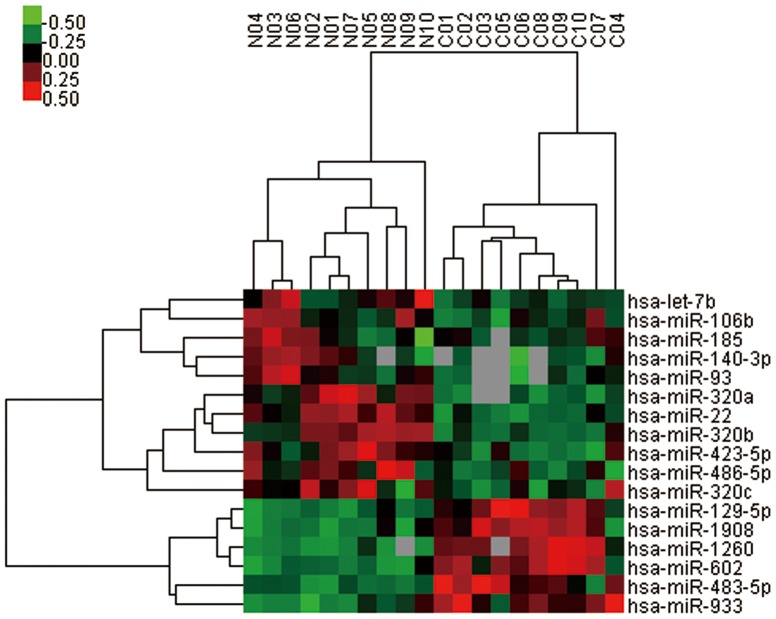
Circulating miRNAs signature in CTEPH patients and healthy controls. Heirarchical clustering and heatmap diagram of the optimum circulating miRNAs signature derived from the greedy search. RNA was isolated from EDTA-coagulated plasma from CTEPH patients (n = 10) and healthy controls (n = 10).

To further determine the molecular signature of CTEPH, the greedy search was applied. Based on the 35 differentially expressed miRNAs from the microarray, we identified a 17 miRNA combination showing the best diagnostic efficacy. The diagnostic models constructed with them gave diagnostic sensitivity and specificity of >0.9 for all the seven methods (Table S4 in File S1). Hierarchical cluster of miRNAs in the signature was shown in Figure 1. Among the included miRNAs, the top five of the six up-regulated miRNAs were miR-1260, miR-602, miR-129-5p, miR-1908 and miR-483-5p by foldchange, while the top five of eleven down-regulated miRNAs were miR-140-3p, miR-93, miR-22, miR-106b and let-7b.

### Predictive Investigation of MiRNA Signature Functions

To illustrate the potential role of differentially expressed miRNAs in pathogenesis of the disease, pathway analysis and GO analysis were applied, and the significant pathways and the top 10% significant GO categories aligned by weight were shown in Figure S1 and Figure S2.

Taking the possible pathophysiology of CTEPH into account, we filtered these significant pathways and GO categories for the most important functions. As displayed in Table 2, the pathways associated with proliferation were the most important ones, and the pathways associated with immunity were up-regulated, while some cardiovascular related pathways were down-regulated. In the GO categories (Table 3), the cell function regulation categories were up-regulated, while the calcium transport regulation and negative regulation of cell proliferation categories were down-regulated.

**Table 2:** Filtered pathways for potential pathogenesis of CTEPH.

Functions	KEGG pathway	Regulated	Weight (-lg*P*)
**Associated with proliferation**	MAPK signaling pathway	up/down	14.51/7.07
	ErbB signaling pathway	up/down	7.19/5.52
	TGF-β signaling pathway	up/down	6.67/4.67
	Wnt signaling pathway	up/down	2.32/2.90
	mTOR signaling pathway	up/down	5.43/4.03
	pathways in cancer	up/down	12.49/3.84
**Associated with immunity**	HTLV-1 infection	up	6.03
	T cell receptor signaling pathway	up	5.06
	Fc gamma R-mediated phagocytosis	up	4.47
	Bacterial invasion of epithelial cells	up	3.37
	Influenza A	up	2.48
	B cell receptor signaling pathway	up	2.43
**Cardiovascular**	Arrhythmogenic right ventricular cardiomyopathy	down	4.10
	Hypertrophic cardiomyopathy	down	3.77
	Vascular smooth muscle contraction	down	3.66
	Dilated cardiomyopathy	down	3.49
	Calcium signaling pathway	down	3.04
	Regulation of actin cytoskeleton	down	2.52

**Table 3:** Filtered GO categories for potential pathogenesis of CTEPH.

Function	GO category	Regulated	Weight (-lg*P*)
**Cell functions**	cell cycle	up	15.50
	cell differentiation	up	11.57
	cell adhesion	up	10.16
	cell division	up	8.86
	cell cycle arrest	up	6.69
	cell migration	up	6.51
**Calcium signal and proliferation**	regulation of calcium ion transport via voltage-gated calcium channel activity	down	5.22
	negative regulation of cell proliferation	down	4.94

### Circulating Let-7b Significantly Decreased in CTEPH Patients

**Figure 2:**
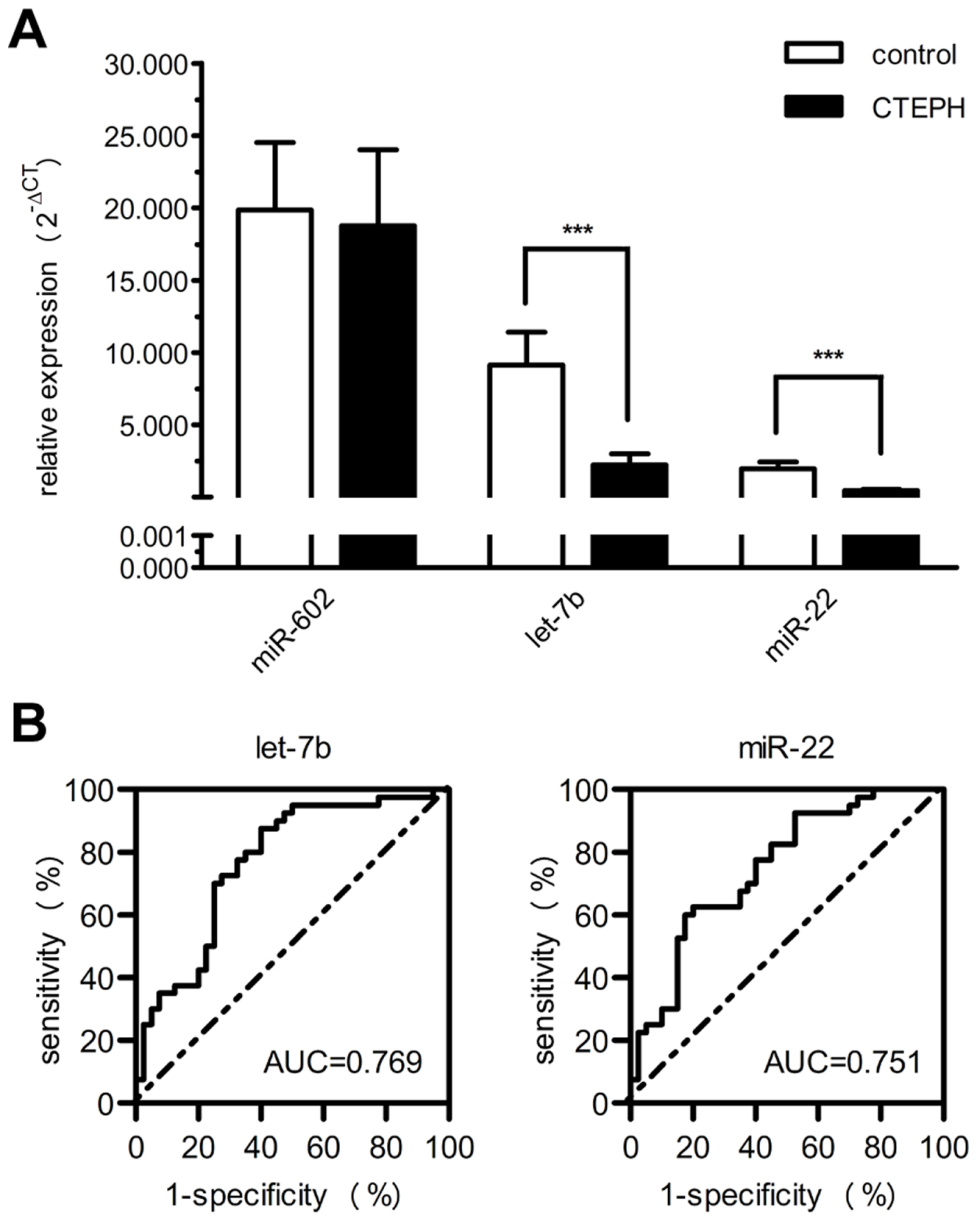
Independent validation of differentially expressed miRNAs. Three candidate miRNAs (miR-602, let-7b and miR-22) from the microarray were validated by qRT-PCR in an independent cohort of CTEPH patients (n = 40) and healthy controls (n = 40). (**A**) Relative expression (2^-ΔCT^) normalized to cel-miR-39 was shown as median with interquartile. *P* value was calculated by Mann-Whitney *U* test. ***P<0.001. (**B**) ROC curve analysis and AUC for 2 validated miRNAs (let-7b and miR-22) in the diagnosis of CTEPH.

To confirm the findings obtained from the miRNA profile and attempt to find a candidate miRNA for further investigation on its role in the disease, after a detailed literature review (Table S5 in File S1), we selectively measured the expression of 3 miRNAs in an independent enlarged cohort of CTEPH patients (n = 40) and healthy controls (n = 40) using TaqMan qRT-PCR. Among the 3 included miRNAs, up-regulated miR-602 was oncogenic one, while down-regulated let-7b and miR-22 were anti-oncogenic ones involved in lung disease or cell migration and proliferation (Table S5 in File S1). As shown in Figure 2A, consistent with miRNA profile, let-7b and miR-22 levels were significantly reduced in CTEPH patient plasma (*P*<0.001). However, the up-regulation of miR-602 in the signature was not observed in the validation cohort. In CTEPH patients of validation cohort, the let-7b level was different between patients of positive and negative acute vascular reaction, while positively correlated with the level of plasminogen activator inhibitor 1 and D-Dimer, and inversely correlated with cardiac index (Table S6 in File S1 and Figure S3). ROC curve analysis revealed that let-7b and miR-22 had moderate value for CTEPH diagnosis with the area under the curve of 0.769 (95% CI: 0.664–0.874) and 0.751 (95% CI: 0.645–0.857) respectively (Figure 2B).

### ET-1 and TGFBR1 Were Direct Targets of Let-7b

In order to study the function of validated miRNAs in CTEPH, first we predicted their target genes by *in silico* analysis. The results showed that ET-1 and many important receptors in TGF-β and mitogen-activated protein kinase pathways contained let-7b binding sites in their 3′-UTRs, suggesting that they might be potential targets of let-7b. Thus we selected let-7b as the candidate miRNA in the following studies. The most highly targeted pathways and the potential targets of let-7b included in these pathways were displayed in Table S7 in File S1. Among the top 10 pathways, TGFBR1 was included in five of them, and this result suggested that TGFBR1 might be a core target of let-7b.

**Figure 3:**
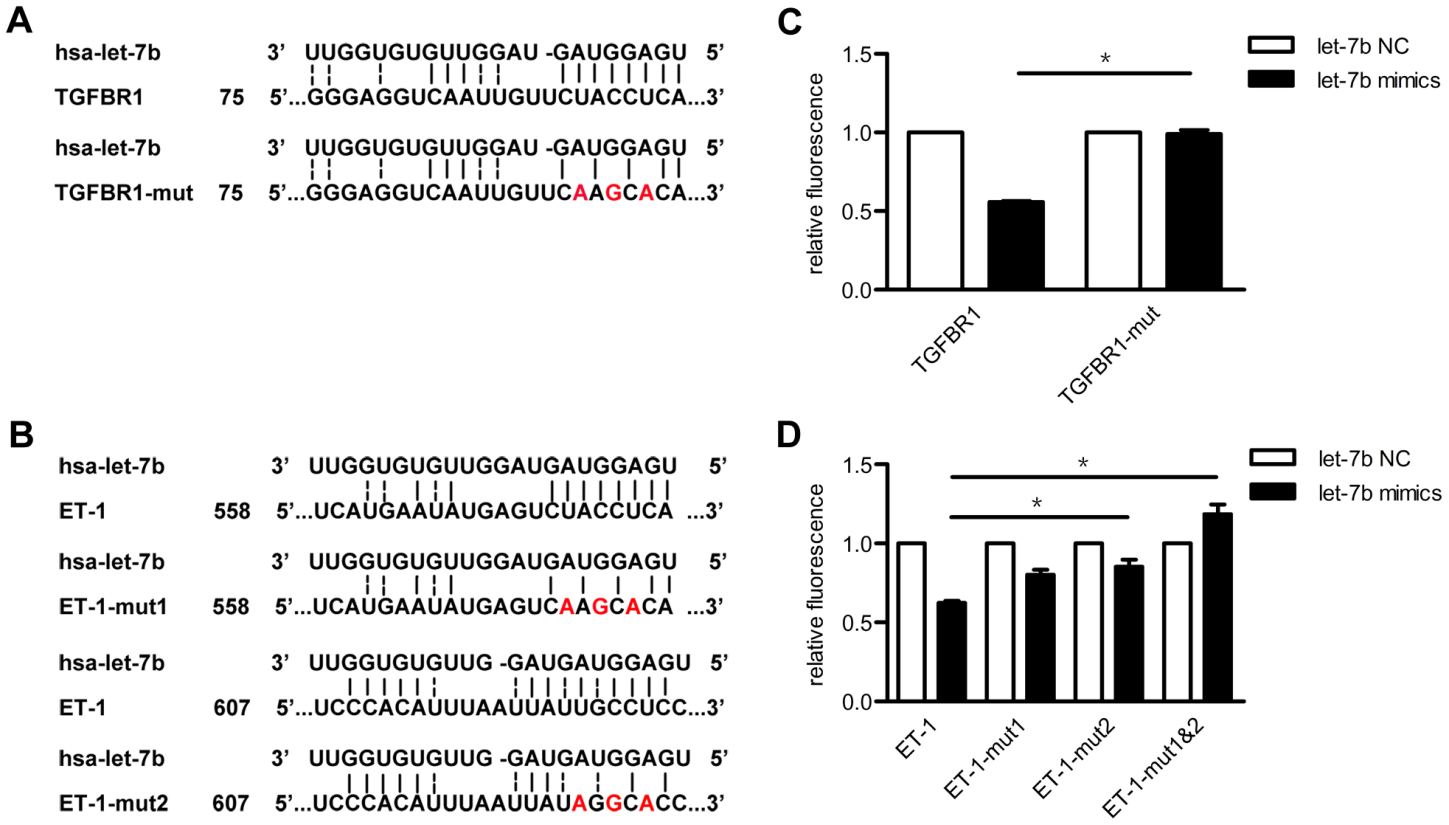
ET-1 and TGFBR1 were direct target genes of let-7b. EGFP reporter assay was applied for target validation in HEK 293 cells. Oligonucleotide mimics and negative control (NC) of let-7b were co-transfected with reporter plasmids and mutated controls, respectively. (**A**) **&** (**B**) Predicted duplex of let-7b and its target region of TGFBR1 and ET-1 with experimental mutation in seed sequences respectively. The number indicates the region position in its 3′-UTRs. The mutated nucleotide was shown in red, and the original nucleotide was its complementary one. (**C**) **&** (**D**) let-7b down-regulated EGFP expression through the predicted seed sequences in 3′-UTRs of ET-1 and TGFBR1. *P* value was calculated by two-sample Kolmogorov-Smirnov test. * *P*<0.05.

To determine whether let-7b directly targets ET-1 or TGFBR1 as predicted, we constructed an EGFP reporter carrying the putative let-7b binding sites in 3′-UTR of ET-1 or TGFBR1. Control constructs were generated from the wild ones by point mutation respectively (Figure 3A and 3B). Co-transfection of HEK293 cells with wild-type 3′-UTR TGFBR1 reporter constructs and 2′-OME modified let-7b mimics resulted in an approximately 45% reduction of EGFP fluorescence compared with control transfection (Figure 3C). The reduction was blunted when the seed binding site was mutated (Figure 3C). As predicted by Targetscan and RNAhybrid, there were two let-7b binding sites in the 3′-UTR of ET-1 (Figure 3B). Approximately 40% reduction of EGFP fluorescence with a construct containing a wild type 3′-UTR of ET-1 was detected compared to control transfection with all binding sites mutated construct(Figure 3D). The binding site, which was predicted by RNAhybrid (ET-1-mut2), seemed to be more powerful than the other one. These data suggested that TGFBR1 and ET-1 were direct targets of let-7b.

### Let-7b Was Inversely Correlated with Plasma ET-1 Level in CTEPH Patients

**Figure 4:**
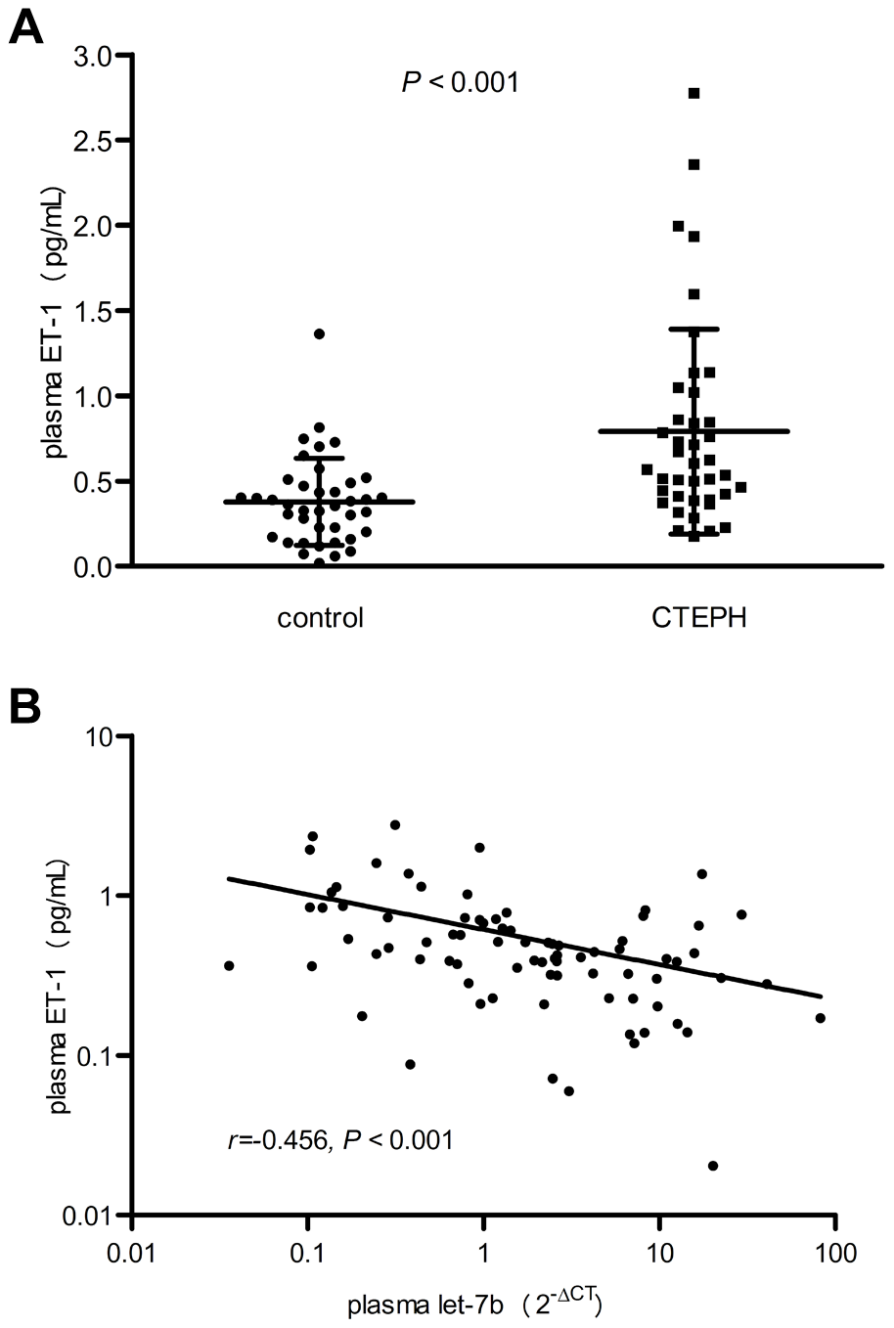
Elevated plasma ET-1 level in CTEPH patients and its correlation with let-7b. (**A**) Plasma endothelin-1 level of CTEPH patients (n = 40) and healthy controls (n = 40) measured by ELISA. *P* value was calculated by Mann-Whitney *U* test. *P*<0.001. (**B**) Spearman correlation and scatter plot of plasma endothelin-1 and let-7b (n = 80). The X- and Y-axis were log10 transformed. The correlation coefficient and *P* value were shown.

As demonstrated above, ET-1 was one of the direct targets of let-7b. Literature showed that ET-1 was the key vasoconstrictor of pulmonary circulation, and has been indicated in the pathophysiology of pulmonary hypertension including CTEPH [18]. To further determine the association between ET-1 expression and let-7b, the plasma ET-1 level of the validation cohort was detected by ELISA. As shown in Figure 4A, the plasma ET-1 level was significantly elevated in CTEPH patients compared with healthy controls (*P*<0.001). This was consistent with previous report [18]. Furthermore, the ET-1 level was inversely related to the plasma let-7b level moderately (*r* = −0.456, *P*<0.001) (Figure 4B). These data suggested that the down-regulation of let-7b might be related to the elevated ET-1 level of CTEPH patients.

### Altering Let-7b Regulated ET-1 and TGFBR1 Expression in PAECs

**Figure 5:**
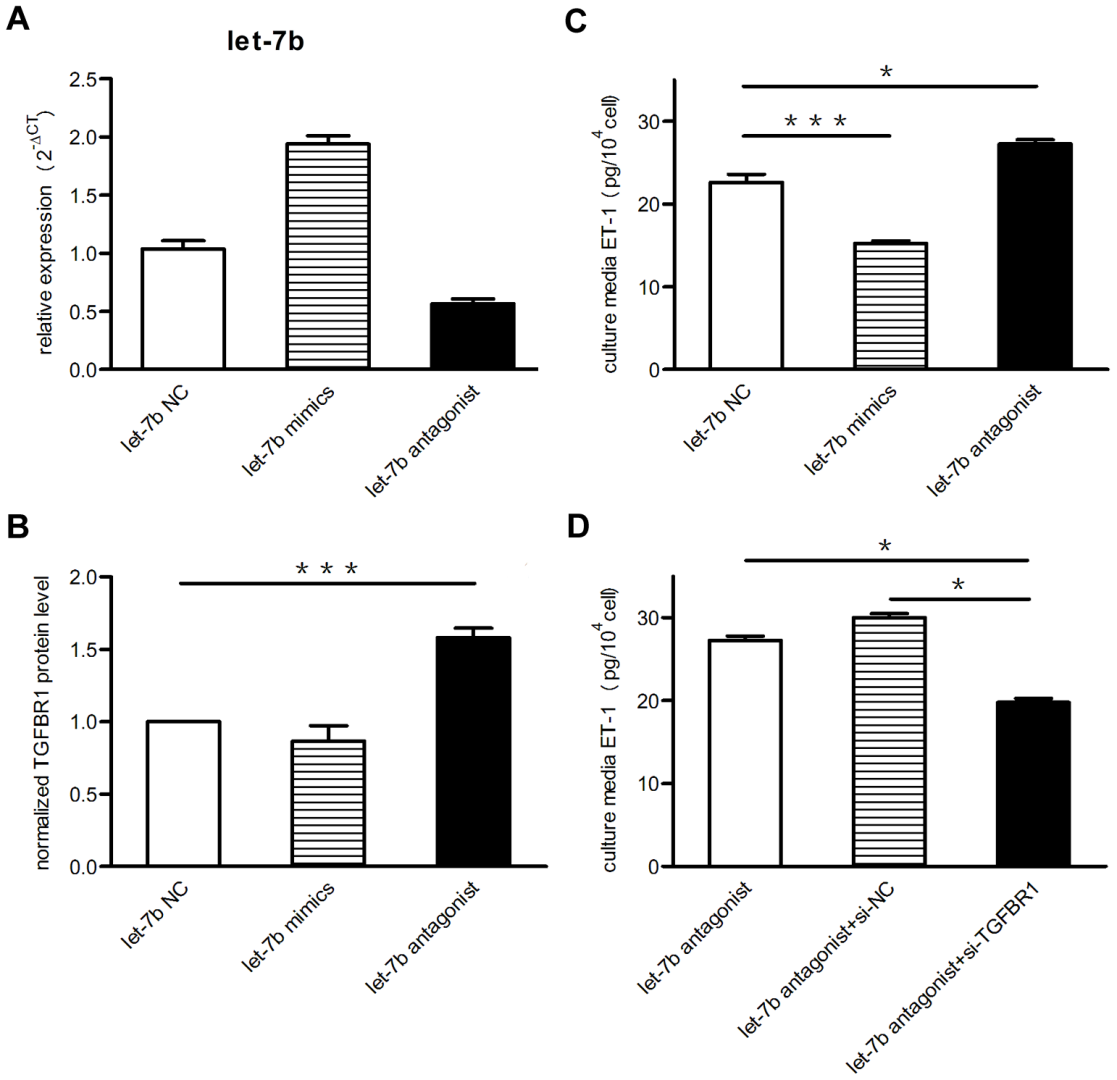
Altering let-7b regulated ET-1 and TGFBR1 expression in PAECs. Human pulmonary arterial endothelial cells (PAECs) were infected with a let-7b antagonist/\mimics lentivirus or empty control. (**A**) Endogenous let-7b was decreased to a level about 55% by antagonist lentivirus and was increased to about 2 fold by mimics. (**B**) After normalized by β-actin, TGFBR1 expression was increased about a half in let-7b antagonist infected PAECs and slightly decreased in let-7b over-expressed cells by western blotting. (**C**) Culture medium was collected after continuous culture for 48 h, and ET-1 levels were detected by ELISA. ET-1 level in culture medium of let-7b over-expressed PAECs was obviously decreased, and was increased in let-7b antagonized PAECs. (**D**) Let-7b antagonized cells were transfected with siRNA for TGFBR1 and control siRNA respectively. Silencing TGFBR1 with siRNA could reverse the increased ET-1 level causing by let-7b antagonist (n = 5). *P* value was calculated by two-sample Kolmogorov-Smirnov test. *** *P*<0.001. * *P*<0.05.

To further illustrate the function of let-7b, let-7b antagonist lentivirus or let-7b mimics were used to decrease or increase the let-7b expression in human PAECs. A multiplicity of infection of 20 achieved an infection rate of more than 90% for antagonist lentivirus and 80%–90% for let-7b mimics (data not shown), and the let-7b level was decreased approximately 45% and increased for 2 fold (Figure 5A). In the let-7b antagonized PAECs, TGFBR1 expression increased remarkably, and the reverse results were seen in let-7b over-expressed cells (Figure 5B). Similar results were also found for ET-1 level in the culture medium of PAECs (Figure 5C). ET-1 expression is a complex process regulated by multiple cytokines, including TGF-β. Previous studies showed that TGF-β stimulated expression of ET-1 preferentially through TGFBR1 [30]. As let-7b can also target TGFBR1, the effect of TGFBR1 on the let-7b regulation of ET-1 should be clarified. Thus, we silenced TGFBR1 in let-7b antagonized PAECs with siRNA. As shown in Figure 5D, the increased ET-1 level of let-7b antagonized PAECs was reversed to approximately normal level. These data indicated that TGFBR1 was involved in the let-7b regulation of ET-1, and this seemed to be over the effect of direct targeting.

### Let-7b Regulated PAECs and PASMCs Migration through TGFBR1 and ET-1

**Figure 6:**
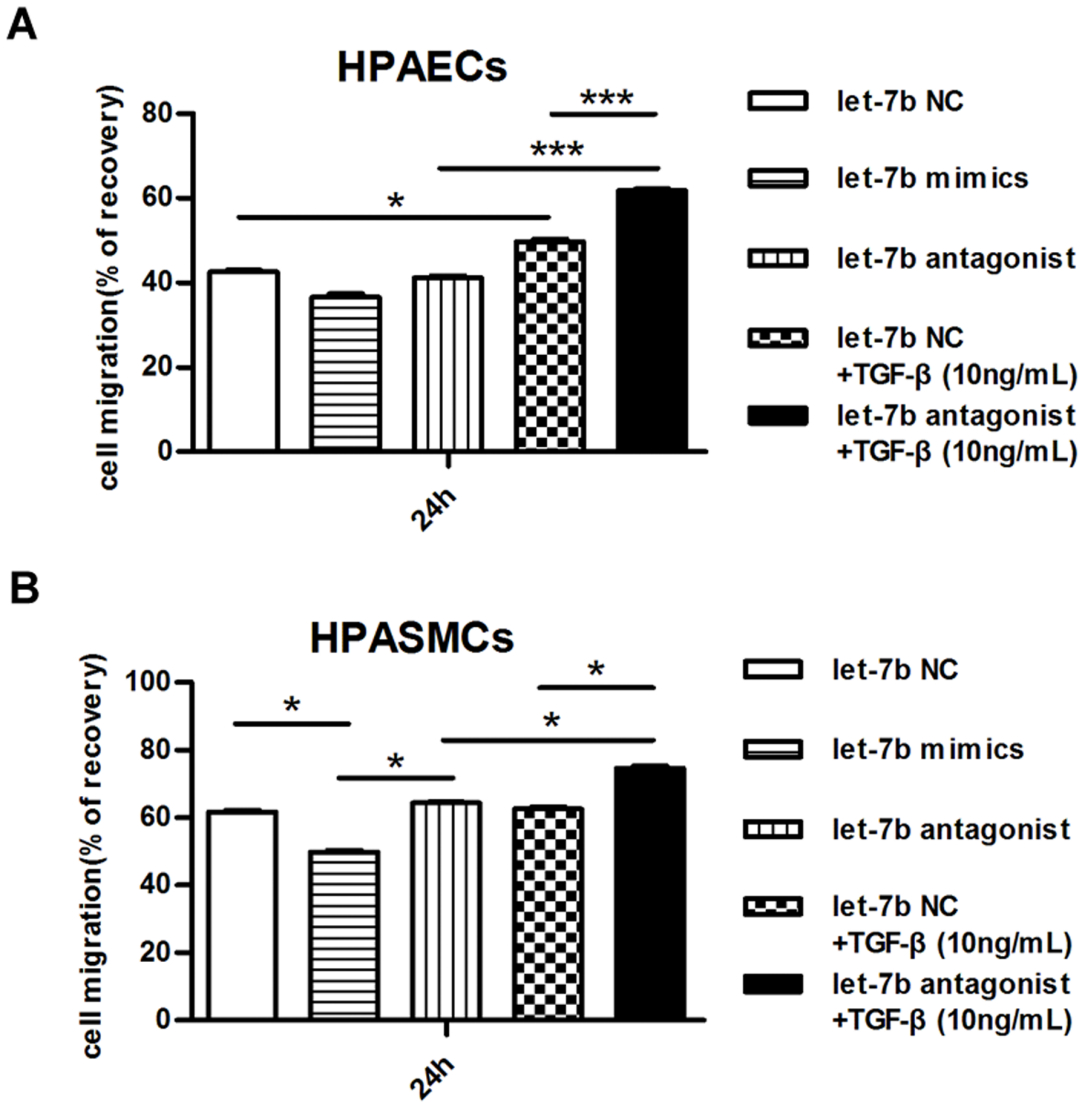
Let-7b regulated PAECs and PASMCs migration. Human PAECs or PASMCs were transfected with a let-7b antagonist lentivirus or let-7b mimics, and the migration was evaluated by wound healing assays. Pictures were taken at 0 h, 12 h, 24 h, and 48 h. The wounded area was expressed as the percentage of recovery. (**A**) Let-7b mimics suppressed PAECs migration, and its antagonist promoted TGF-β induced PAECs migration at 24 h (n = 5). (**B**).Let-7b mimics suppressed PASMCs migration, and its antagonist promoted TGF-β induced PASMCs migration at 24 h (n = 5). *P* value was calculated by two-way ANOVA, and Post Hoc Test was done by Student-Newman-Keuls method. *** *P*<0.001. * *P*<0.05.

Pulmonary vascular remodeling is the crucial pathological change in CTEPH, and activated migration of PAECs and/or PASMCs contributes to this process. We investigated the possible role of let-7b in migration of PAECs and PASMCs by wound healing assay after decreasing or increasing its expression using the antagonist lentivirus or let-7b mimics respectively as Figure 5. The PASMCs showed the similar efficiency as PAECs did, which was shown in Figure 5A. After Post Hoc Test, as shown in Figure 6A, migration of PAECs was slighted inhibited when let-7b was over-expressed, but antagonist showed no effect when PAECs were cultured in normal medium. For TGFBR1 and ET-1 have been identified as direct targets of let-7b, we supposed that they might participated in let-7b regulated cell migration. Then TGF-β (10 ng/mL) was added into the culture media. The results showed that TGF-β could induce the migration of let-7b NC PAECs, while when cultured in medium with TGF-β, migration of let-7b antagonized PAECs was further promoted. Similar results were observed for PASMCs except for induction of TGF-β on let-7b PASMCs (Figure 6B). The results above indicated the involvement of TGFBR1 in let-7b regulated migration of PASMCs and PAECs.

For ET-1, because there was no pro-migration effect observed by let-7b antagonist induced ET-1 increase (Figure 5C and Figure 6A), a selective endothelin A receptor antagonist (ETA, BQ-123, Sigma Aldrich, St. Louis, US) was used to observe the effect of derepression of ET-1 on migration of PAECs. As shown in Figure S4A, ETA (1 nM) could suppress normal PAECs migration, and the suppression effect was weakened by let-7b antagonist. For PASMCs, as they were not the chief cells for endogenous ET-1 expression, exogenous ET-1 was used to observe its effect on PASMCs migration. And we saw that ET-1 could promote the migration of both let-7b NC and let-7b antagonized PASMCs (Figure S4B). These results above indicated that although ET-1 was not the major mediator between let-7b and PAECs migration, derepression of ET-1 by let-7b partially participated in the PAECs migration, and the elevated ET-1 also could induce PASMCs migration.

## Discussion

In present study, we demonstrated that CTEPH patients had a differently expressed miRNA profile. And a signature of 17 miRNAs was shown to be related to the disease pathogenesis and gave the diagnostic efficacy of both sensitivity and specificity >0.9. Let-7b, one of the key miRNAs, might be involved in the pathogenesis of CTEPH by affecting ET-1 expression and the migration of PAECs and PASMCs.

Even though many studies have focused on the associated risk factors, genetic susceptibility, pathology, therapy, prognosis for CTEPH [2], [7], [10], [31] in recent years, still there is much to be further recognized, especially for its pathogenesis. Early treatment before right heart insufficient by pulmonary endarterectomy or suitable medical treatment was crucial for prognosis improvement [32]. This highlighted the need to develop sensitive and reliable biomarkers for early diagnosis of CTEPH.

At present, possibly owing to the complex pathophysiology of CTEPH, the reported candidate molecular biomarkers for CTEPH, including asymmetric dimethylarginine [33], D-Dimer [34], heart-type fatty acid-binding protein [35] and brain natriuretic peptide [36] still were not sufficiently reliable for clinical application. Therefore, combination of some biomarkers representing different pathophysiological aspects of the disease might be the tendency for future biomarker screening. The emerging of microarray technology has made it possible to achieve tens of thousands of gene expression simutaneously as the base of screening. Biomarker signature have been studied in many diseases [37], [38], including cancers, cardiovascular diseases. MiRNAs were recognized of limited amount to regulate most of protein-coding gene expression post-transcriptionally. Therefore, it seemed more practical to explore miRNAs as biomarkers for the diseases with complex etiology and pathophysiology. Studies on miRNA signature have increased steeply during recent few years [39], [40]. These studies have shown that certain miRNA signature had satisfactory efficacy for disease diagnosis and evaluation.

As ideal diagnostic biomarker, miRNAs possess many sustaining properties. First, circulating miRNAs were remarkably stable even exposed to harsh environment [15], [24] and this characteristics made detection reproducibly. Second, the pathognostic cell resource of miRNAs [14] determined the high specificity of circulating miRNAs for disease diagnosis. Third, miRNAs were small molecules that lacked post-processing modification. They could be detected by an extremely sensitive method at very low starting concentration [41]. Although large population verification of the results in our study is still needed, circulating miRNAs provide a promising prospect for CTEPH diagnosis. During verification, the signature can be further simplified for clinical application, and the efficacy and accuracy can be enhanced.

Since 2008, many studies have indicated that circulating miRNAs were functional molecules which might act in cell communication and suppress the translation of target genes in recipient cells. A recent study [15] showed that, in healthy subjects, circulating miRNA profile was similar to the profile of circulating blood cells, but this similarity was disturbed in diseased subjects, and the circulating miRNA profile was endued with characteristics of cells involved in the disease. In this study, we observed that the differentially expressed circulating miRNAs had important regulatory function in CTEPH pathogenesis, such as remodeling of pulmonary vascular, imbalance of vascular tone or inflammation. Many of these were regarded to be involved in CTEPH etiology. However, few of them have been thoroughly interpreted. Functions regulated by the aberrant miRNA signature were the cue for the comprehensive understanding of CTEPH pathogenesis. Based on these *in silico* results, we selectively focused on let-7b to study its cellular function relevant to the disease, because the predicted targets of it were likely to be involved in CTEPH pathogenesis. Let-7b has been reported to be an anti-oncogenic miRNA which is frequently lost in many tumors [13], [42]. By the preliminary correlation analysis with clinical characteristics of CTEPH patients, we found that circulating let-7b levels were decreased in patients with negative AVR, which was generally regarded to be related with serious pulmonary vascular remodeling. This illuminating result indicated the possible role of let-7b in pulmonary vascular biology of CTEPH pathogenesis. In the further mechanism study, we found that let-7b could target ET-1 and TGFBR1, which have been reported to be closely related to the pathogenesis of CTEPH. Antagonizing let-7b could up-regulate ET-1 and TGFBR1 expression, which promoted PAECs and PASMCs migration. These effects would lead to persistent constriction or remodeling of pulmonary vascular bed and promote CTEPH development. Positive correlation was also found between let-7b and PAI-1 or D-Dimer, which were regarded to be elevated in thrombotic diseases. Therefore, the positive correlation gave no direct indication for the role of let-7b in coagulation process of CTEPH. The exact mechanism still needs further study.

ET-1 was a potent endothelium-derived vasoconstrictor [43]. It was mainly secreted by endothelial cells and mediate vascular constriction and PASMCs proliferation through endothelin A and B receptors [44], [45]. In CTEPH patients, increases of ET-1 were significantly correlated with clinical characteristics [10]. In addition, elevated serum ET-1 was demonstrated to be a predictor of bad pulmonary endarterectomy outcome [46]. Endothelin receptor antagonists have emerged as cornerstone treatment for PAH for more than 10 years [47]. In CTEPH patients, especially inoperable ones, ETAs were also of benefit in hemodynamics [20]. ET-1 expression was a complex biological process. In the present study, we showed a new aspect of ET-1 expression regulation at the post-transcriptional level by a miRNA. The down-regulation of let-7b was correlated with elevation of plasma ET-1 level, and this might be accomplished through two ways. First, ET-1 was a direct target of let-7b, and it was derepressed when let-7b was down-regulated. Second, TGF-β was one of the most potent regulators of ET-1 expression [48]. It strongly increased ET-1 mRNA and protein expression in endothelial, and specifically, TGF-β induced ET-1 expression preferentially through the TGFBR1/Smad3 pathway [30], [49]. Our results suggested that decreased let-7b up-regulated the expression of TGFBR1, which was in turn possibly involved in the elevation of ET-1 in CTEPH patients. In addition, ET-1 is a mitogenic growth factor especially in pulmonary circulation. By wound healing assay, we further illustrated that derepression of ET-1 by let-7b partially participated in the PAECs migration, and the elevated ET-1 could induce PASMCs migration. The aberrant migration of PAECs and PASMCs was further related to the pulmonary vascular remodeling of CTEPH patients.

Besides regulation of ET-1 expression, TGFBR1 and downstream signals played an important role in biology of pulmonary vessels. In monocrotaline induced pulmonary hypertension rat model, TGFBR1 was highly expressed in the lung [11]. And in isolated PASMCs of PAH patients, TGFBR1 was activated and implicated in pro-proliferative and anti-apoptotic phenotype of them [21]. In a study on TGF-β1 and related receptors of peripheral blood leucocytes, the TβRI/TβRII ratio was significant increased in PAH [50]. These studies indicated an activated status of TGFBR1 during pulmonary hypertension, and in this study, we provide an indirect evidence for a potential role of TGFBR1 in CTEPH via let-7b. Up-regulation of TGFBR1 caused by let-7b, despite other receptors in TGF-β pathway, might lead to the proliferative vascular disease. During the experiment, we found a phenomenon that even with no effect on promoting PASMCs migration either by TGF-β or let-7b antagonist alone, the combinatorial treatment of them obviously promoted the migration of PASMCs. And we supposed the possible reason as follow: first, previous studies showed that TGF-β had dual role in vascular smooth muscle cell on phenotypic transformation [51], [52]. It not only induced SMCs differentiation to the contractile phenotype, but also promoted SMCs dedifferentiation and induced the vascular remodeling. So we supposed that this phenomenon might just be a possible performance of normal PASMCs under TGF-β treatment. Second, TGFBR1 up-regulation has been reported to be related with pulmonary hypertension [11], [21], [50], and we have indentified that TGFBR1 was a direct target of let-7b. Thus we supposed that let-7b antagonist might be involved in cell migration by regulating TGFBR1 expression. As the target of let-7b was not TGF-β itself, let-7b antagonist alone did not induced PASMC migration without exogenous TGF-β. While under combinatorial treatment of let-7b antagonist and TGF-β, the derepressed TGFBR1 induced by let-7b antagonist was highlighted by its ligand TGF-β, and it led to the aberrant migration observed in let-7b antagonist PASMCs.

There are still some limitations in our study. First, these results were derived from relative small population, and the signature still needed to be validated and adjusted. Second, as miRNAs existing in plasma are in relative low concentration, some low abundance miRNAs might not be reliably detected in our microarray cohort. This might lead to some selection bias of miRNAs for pathogenesis study of CTEPH. However, as CTEPH is a multiple etiology disease, diverse sources of the circulating miRNA profile might be more advantageous for understanding the disease compared with relying on single profiles from single diseased tissue or cell. Third, regulation by miRNAs occurs at the network level. In this study, we selectively investigated the function of let-7b, but other miRNAs that contributed to the disease and their communication with let-7b, especially for the up-stream signal of let-7b and its role in CTEPH, still needed further study and evaluation. Fourth, our previous study of miRNA profile of PASMCs culture from pulmonary endarterectomy tissue also found let-7d, another member of let-7 family, was decreased and involved in the cell proliferation [9], which indicated the importance of let-7 family in pathogenesis of CTEPH. A hypothesis about the mechanism leading to the decrease of let-7 family is still needed to be set in our further study. We also should pay more attention to its possible effect in view of a future cure.

To our best knowledge, this is the first report of circulating miRNA profile of CTEPH. The results of this study provided us some clue and candidate for further pathogenesis investigation and clinical biomarker screening of this miscellaneous disease.

## Supporting Information

Figure S1 **The significant targeted pathways of miRNAs in the signature.** Seventeen candidate miRNAs in the signature were included in target prediction and KEGG pathway analysis. The significance was defined with *P*<0.05, and the pathways were ranked by the weight (-lg*P*). (**A**) Significant targetgene related pathways of up-regulated miRNAs in CTEPH patients. (**B**) Significant targetgene related pathways of down-regulated miRNAs in CTEPH patients.
(TIF)

Figure S2 **The top 10% significant targeted GO categories of miRNAs in the signature.** Seventeen candidate miRNAs in the signature were included in target prediction and GO analysis. The significance was defined with *P*<0.05, and the top 10% significant GO categories were ranked by the weight (-lg*P*). (**A**) Top 10% significant targetgene related GO categories of up-regulated miRNAs in CTEPH patients. (**B**) Top 10% significant targetgene related pathways of down-regulated miRNAs in CTEPH patients.
(TIF)

Figure S3 **Scatter plots of circulating miRNA concentrations of CTEPH patients against common clinical characteristics.** The two validated candidate miRNAs were analyzed, and the index with statistical significance was displayed. (**A**) Plasma let-7b against CI (n = 35). (**B**) Plasma let-7b against PAI-I (n = 19). (**C**) Plasma let-7b against D-Dimer (n = 37). (**D**) Plasma miR-22 against total homocysteine (tHcy) (n = 28). (**E**) Plasma miR-22 against CI (n = 35). (**F**) Plasma miR-22 against PAI-I (n = 19). (**G**) Plasma miR-22 against D-Dimer (n = 37). (**H**) Plasma miR-22 against CRP (n = 29).
(TIF)

Figure S4 **Let-7b regulated PASMCs/PAECs migration partially through ET-1.** The two validated candidate miRNAs were analyzed, and the index with statistical significance was displayed. (**A**) Transfected PAECs was treated with endothelin A receptor antagonist (ETA, 1nM). ETA could inhibit control PAECs migration, and the inhibition role was weakened by let-7b antagonist (n = 5). (**B**) Normal and let-7b antagonized PASMCs were treated by ET-1 (1 nM). ET-1 could obviously promote migration of both cells, and no difference was observed between the promotion of both cells (n = 5). *P*-value was calculated by two-way ANOVA, and Post Hoc Test was done by Student-Newman-Keuls method. *** *P*<0.001. * *P*<0.05.
(TIF)

File S1 **Supporting materials and methods and tables.** Table S1, Sequences of the primers used in fluorescent reporter assay. Table S2, Differences in clinical characteristics between microarray cohort and validation cohort. Table S3, Properties of miRNAs of CTEPH patients and healthy controls differentially expressed in microarray. Table S4, Diagnostic efficacy of seven methods for the 17 miRNA signature. Table S5, Literature review for the functions of candidate miRNA in the signature. Table S6, Differences of circulating miRNA levels between different conditions of clinical characteristics. Table S7, Top ten powerfully target pathways of let-7b by DIANA-miRPath.
(DOC)
