# Supplementary figures and images for: Differentially Expressed Plasma MicroRNAs and the Potential Regulatory Function of Let-7b in Chronic Thromboembolic Pulmonary Hypertension
Source: PLoS One. 2014 Jun 30;9(6):e101055. doi: 10.1371/journal.pone.0101055 (PMC4076206; doi:10.1371/journal.pone.0101055)

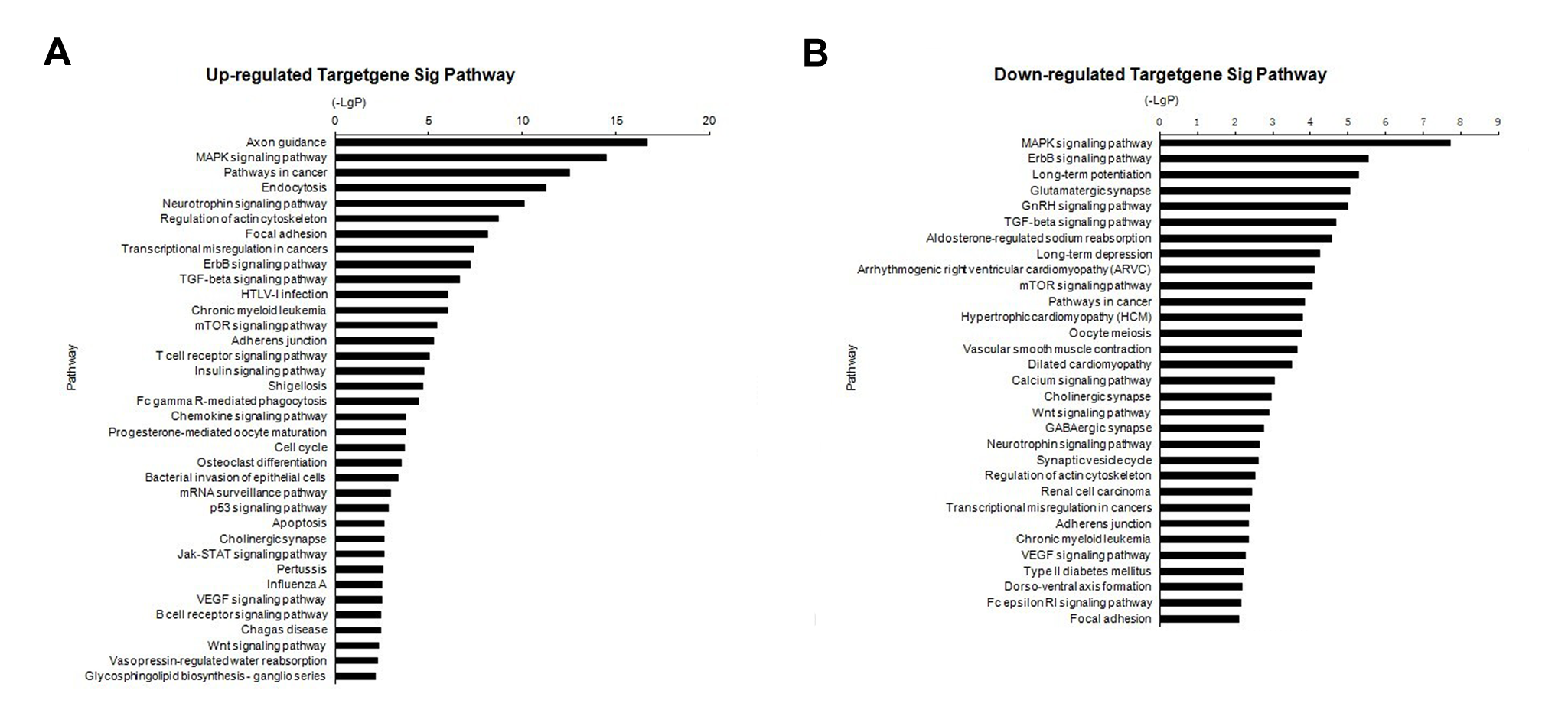

Supplement: Figure S1 — The significant targeted pathways of miRNAs in the signature. Seventeen candidate miRNAs in the signature were included in target prediction and KEGG pathway analysis. The significance was defined with P<0.05, and the pathways were ranked by the weight (-lgP). (A) Significant targetgene related pathways of up-regulated miRNAs in CTEPH patients. (B) Significant targetgene related pathways of down-regulated miRNAs in CTEPH patients. (TIF) [file pone.0101055.s001.tif]

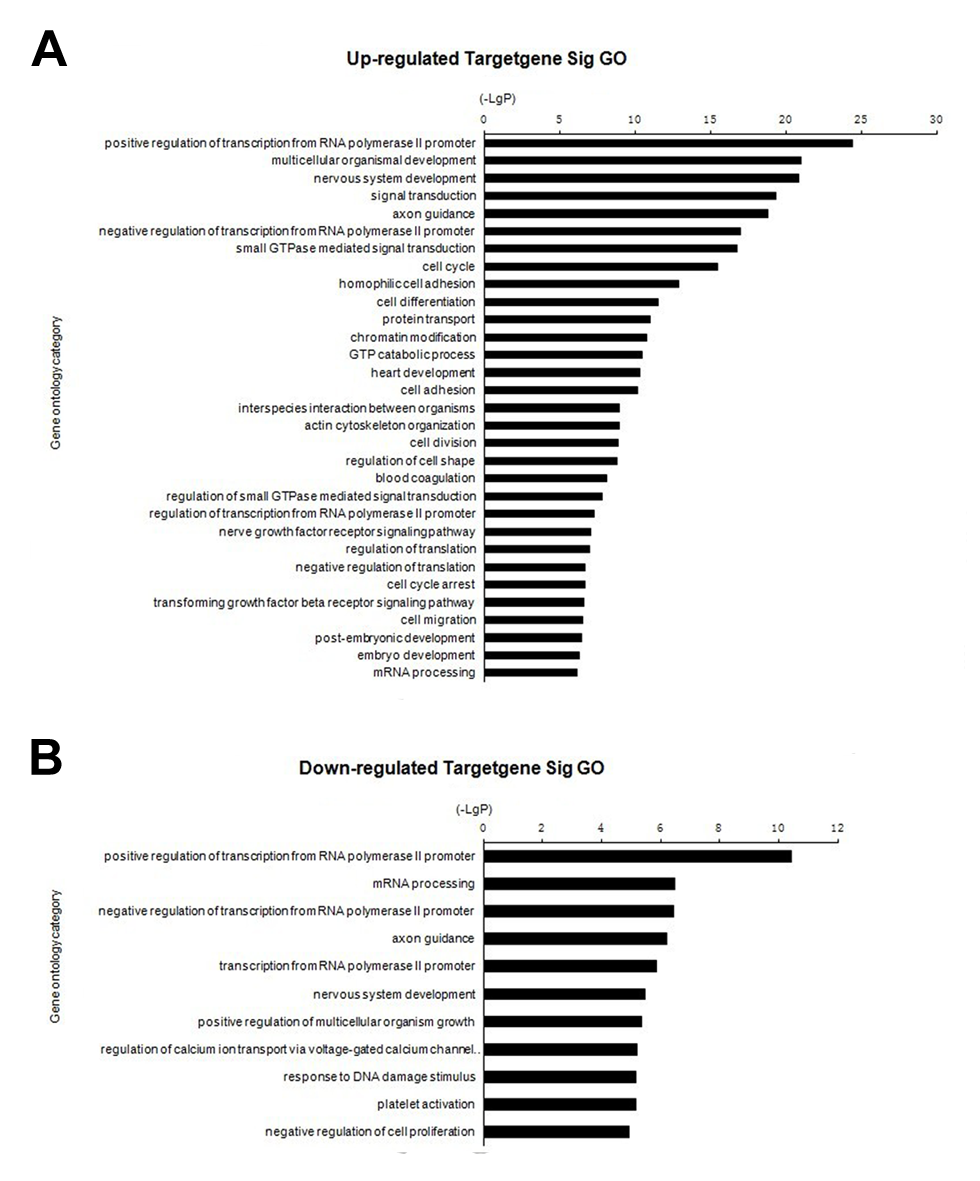

Supplement: Figure S2 — The top 10% significant targeted GO categories of miRNAs in the signature. Seventeen candidate miRNAs in the signature were included in target prediction and GO analysis. The significance was defined with P<0.05, and the top 10% significant GO categories were ranked by the weight (-lgP). (A) Top 10% significant targetgene related GO categories of up-regulated miRNAs in CTEPH patients. (B) Top 10% significant targetgene related pathways of down-regulated miRNAs in CTEPH patients. (TIF) [file pone.0101055.s002.tif]

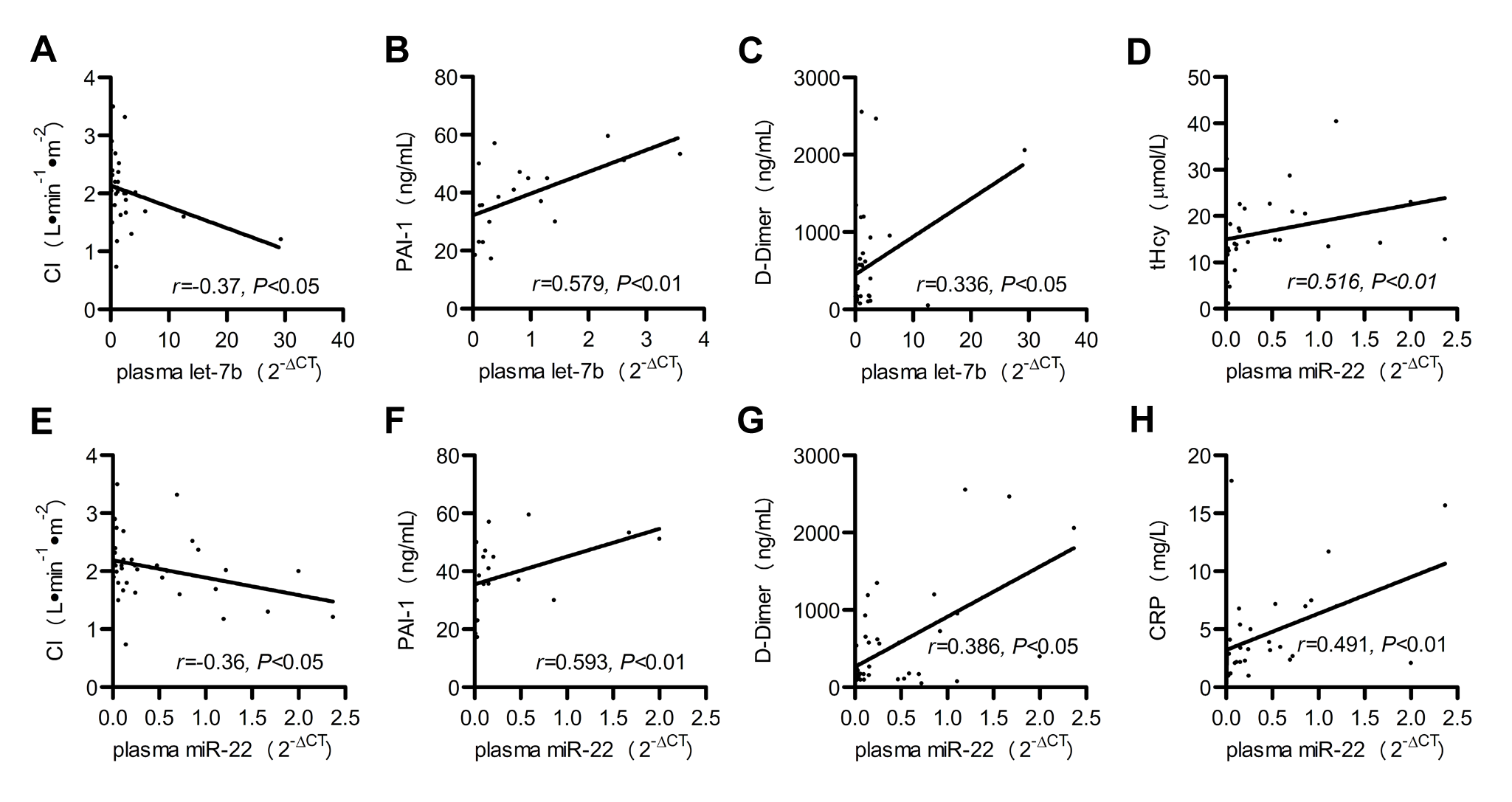

Supplement: Figure S3 — Scatter plots of circulating miRNA concentrations of CTEPH patients against common clinical characteristics. The two validated candidate miRNAs were analyzed, and the index with statistical significance was displayed. (A) Plasma let-7b against CI (n = 35). (B) Plasma let-7b against PAI-I (n = 19). (C) Plasma let-7b against D-Dimer (n = 37). (D) Plasma miR-22 against total homocysteine (tHcy) (n = 28). (E) Plasma miR-22 against CI (n = 35). (F) Plasma miR-22 against PAI-I (n = 19). (G) Plasma miR-22 against D-Dimer (n = 37). (H) Plasma miR-22 against CRP (n = 29). (TIF) [file pone.0101055.s003.tif]

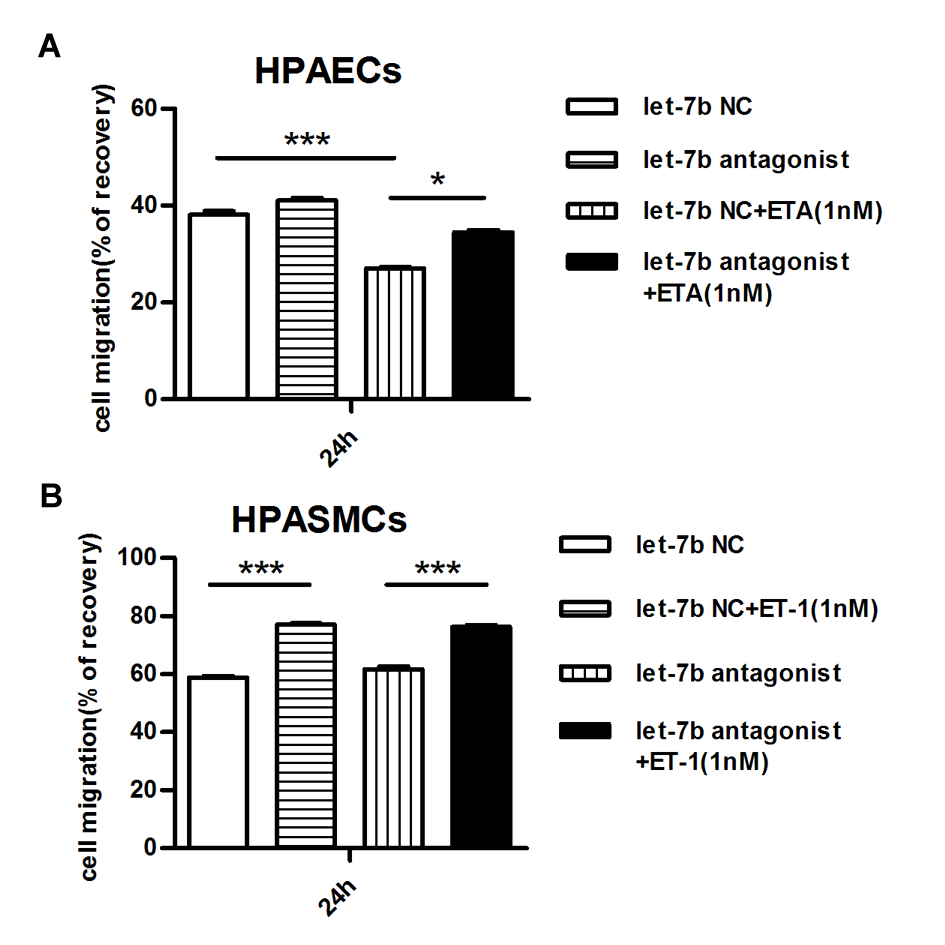

Supplement: Figure S4 — Let-7b regulated PASMCs/PAECs migration partially through ET-1. The two validated candidate miRNAs were analyzed, and the index with statistical significance was displayed. (A) Transfected PAECs was treated with endothelin A receptor antagonist (ETA, 1nM). ETA could inhibit control PAECs migration, and the inhibition role was weakened by let-7b antagonist (n = 5). (B) Normal and let-7b antagonized PASMCs were treated by ET-1 (1 nM). ET-1 could obviously promote migration of both cells, and no difference was observed between the promotion of both cells (n = 5). P-value was calculated by two-way ANOVA, and Post Hoc Test was done by Student-Newman-Keuls method. *** P<0.001. * P<0.05. (TIF) [file pone.0101055.s004.tif]
