# Supplementary material for: Differentially Expressed Plasma MicroRNAs and the Potential Regulatory Function of Let-7b in Chronic Thromboembolic Pulmonary Hypertension
Source: PLoS One. 2014 Jun 30;9(6):e101055. doi: 10.1371/journal.pone.0101055 (PMC4076206; doi:10.1371/journal.pone.0101055)
Supplement: File S1 — Supporting materials and methods and tables. Table S1, Sequences of the primers used in fluorescent reporter assay. Table S2, Differences in clinical characteristics between microarray cohort and validation cohort. Table S3, Properties of miRNAs of CTEPH patients and healthy controls differentially expressed in microarray. Table S4, Diagnostic efficacy of seven methods for the 17 miRNA signature. Table S5, Literature review for the functions of candidate miRNA in the signature. Table S6, Differences of circulating miRNA levels between different conditions of clinical characteristics. Table S7, Top ten powerfully target pathways of let-7b by DIANA-miRPath. (DOC) [file pone.0101055.s005.doc]

**Differentially expressed plasma microRNAs and the potential regulatory function of let-7b in chronic thromboembolic pulmonary hypertension**

Lijuan Guo^a, b^, Yuanhua Yang^a, b^, Jie Liu^c^, Lei Wang^a, b^, Jifeng Li^a, b^, Ying Wang^a^, Yan Liu^d^, Song Gu^d^, Huili Gan^e, f^, Jun Cai^d^, Jason X.-J. Yuan^g^, Jun Wang^a, c, *^, Chen Wang^a, b, h, *^

^a^Beijing Key Laboratory of Respiratory and Pulmonary Circulation Disorders, Beijing Chao-Yang Hospital, Capital Medical University, Beijing, P.R. China.

^b^Beijing Institute of Respiratory Medicine, Capital Medical University, Beijing, P.R. China.

^c^Department of Physiology, Capital Medical University, Beijing, P.R. China.

^d^Department of Cardiology, Beijing Chao-Yang Hospital, Capital Medical University, Beijing, P.R. China.

^e^Department of Cardiac Surgery, Beijing Anzhen Hospital, Capital Medical University, Beijing, P.R. China.

^f^Beijing Institute of Heart, Lung and Vessel Disease, Capital Medical University, Beijing, P.R. China.

^g^Department of Medicine, University of Illinois at Chicago, Chicago, IL, USA.

^h^Department of Beijing Hospital, Ministry of Health, Beijing, P.R. China.

**Supporting Information**

**Materials and Methods**

**Diagnosis of CTEPH**

The diagnosis of CTEPH has been established by medical history, symptoms, pulmonary function tests including arterial blood gas analysis at rest and exercise, Doppler echocardiography, right heart catheterization, ventilation-perfusion scan of the lungs, and computed tomography pulmonary angiogram (CTPA) or magnetic resonance pulmonary angiogram (MRPA) [S1]. A panel of clinicians and radiologists review each case.

**Subjects Selection in Healthy Control Group**

The subjects in control group were selected from the patients, who were hospitalized for surgery of cataract, polyps, or plastic. All the subjects were screened by exclusion criterial, and were evaluated by cardiac ultrasound to exclude pulmonary hypertension. For the second question, we collected the control subjects firstly, and then selected the matched ones in age and sex. If there were more than one subjects matched, we took one of them randomly.

**RNA Extraction**

Total RNA was prepared from human plasma using TRI Reagent (Sigma Aldrich, St. Louis, US). In detail, 200 μL of plasma was added to 750 μL TRI Reagent supplemented with 20 μL 5 nM acetic acid, and the mixture was lysed for 5 min at room temperature, followed by supplement with 200 μL of chloroform and shaking vigorously for 15 s. After resting at room temperature for 2-5 min, the resulting mixture was centrifuge at 12000 rpm for 15 min, and the colorless upper aqueous phase was transferred to a fresh tube. Precipitate RNA from the aqueous phase by mixing with 500 μL isopropanol and store in room temperature for 10 min before centrifuge at 12000 rpm for 10 min. The pellet was washed in 75% ethanol by vortex and centrifuge at 12000 rpm for 5 min. Remove the ethanol and briefly air-dry the RNA pellet. Dissolve the pellet in nuclease-free water and incubate for 10 min at 60 °C. RNA concentration was measured by NanoQuant Plate on infinite M200 (Tecan, Männedorf, CH), and RNA was stored at -80 °C until further processing.

**MiRNA Microarray and *In Silico* Analysis**

RNA was labeled with Exiqon miRCURY Array Power Labeling kit (Hy3) and hybridized on the miRCURY LNA Array (version 14.0, Exiqon, Vedbaek, DK) station. Arrays were scanned on the Axon GenePix 4000B microarray scanner and GenePix pro (version 6.0) was used to read the raw intensity of the images. The intensity of the green signal was calculated after background subtraction, and replicated spots on the same slide were averaged to obtain median intensity.

We used the median normalization method to acquire normalized data (foreground minus background divided by median). The miRNAs which were with an intensity > 0 in at least 5 samples for both groups after background correction were filtered for further analysis. RVM *t*-test was applied to determine the differential miRNAs, for its ability in raising degrees of freedom effectively for this relative small sample. The threshold for significance used to define up-regulation or down-regulation of miRNAs was a value of *P* < 0.05.

For the optimum diagnostic model, greedy search, a general term of heuristic algorithms for making locally optimal choices at each stage with the hope of finding a global optimum, was used to find different marker combinations, and seven diagnostic methods (Compound Covariate Predictor, Diagonal Linear Discriminant Analysis, 1-Nearest Neighbor, 3-Nearest Neighbors, Nearest Centroid, Support Vector Machines and Bayesian Compound Covariate Predictor) were used to diagonal samples for each combination by cross-validation to explore different aspects of data. Support Vector Machines could find the optimal hyperplane that was able to separate the data by projecting them into a high-dimensional space. Compound Covariate Predictor and Diagonal Linear Discriminant Analysis used combinations of different weighted expression ratios for classification, whereas Nearest Neighbor and Nearest Centroid are nonlinear and nonparametric methods [S2-S5].TargetScan (http://www.targetscan.org/) and miRDB (http://mirdb.org/) were combined for target prediction. The Gene Ontology (GO) analysis was applied to analyze the main function of the target genes of differentially expressed miRNAs according to the GO Database (http://www.geneontology.org/) which was the key functional classification of NCBI [S6]. Generally, Fisher’s exact test and $\chi^{2}$ test were used to classify the GO category, and the false discovery rate (FDR) was calculated to correct the *P*-value. We computed *P*-values for the GOs of all the predicted target genes of differential miRNAs. Similarly, pathway analysis was used to find out the significant pathway of the differential genes according to the KEGG PATHWAY Database (<http://www.genome.jp/kegg/pathway.html>) [S7].

Bioinformatics search for potential targets of let-7b was accomplished by intersections of several approved mammalian target prediction programs, including TargetScan, miRDB (http://mirdb.org/), miRanda (http://www.microrna.org/) and RNAhybrid (http://bibiserv.techfak.uni-bielefeld.de/rnahybrid/), and DIANA-mirPath (DIANA-mirPath: Integrating human and mouse microRNAs in pathways) was used to determine the most powerfully targeted pathways of it.

**Cell Culture**

HEK293 cells were cultured in Dulbecco’s Modified Eagle Medium (DMEM, Gibco, California, US), supplemented with 10% (v/v) fetal bovine serum (FBS, Gibco, California, US) and incubated at 37 °C in 5% CO_2_. Human pulmonary artery endothelial cells (PAECs, ScienCell, California, US) were cultured in endothelial cell medium (ECM, ScienCell, California, US) supplemented with 5% (v/v) FBS and 1% ECGS (ScienCell, California, US). Human pulmonary artery smooth muscle cells (PASMCs, ScienCell, California, US) were cultured in smooth muscle cell medium (SMCM, ScienCell, California, US) supplemented with 2% (v/v) FBS and 1% SMCGS (ScienCell, California, US). Cells were passaged when reaching 80-90% confluence using 0.1% trypsin with 0.08% EDTA (Sigma-Aldrich, St. Louis, US). The cells were used for experiments at passage 3 to passage 6, and were serum deprived in FBS and growth factor free medium for 24 hours.

**Fluorescent Reporter Assay**

To construct the enhanced green fluorescent protein (EGFP) reporter plasmid, EGFP was cloned from pEGFP-N1 vector (Clontech, California, US) to pcDNA3.1 (Invitrogen, California, US). The 3’-UTR fragments of ET-1 and TGFBR1 containing the predicted hsa-let-7b binding sites were amplified from the human lung fibroblast cDNA of a health donor, and subcloned into pcDNA3.1/EGFP construct by *Not* I and *Xba* I (NEB, Ipswich, US) subsequently. The control constructs were generated by point mutation in the predicted “seed sequence” of hsa-let-7b using TaKaRa MutanBEST Kit (TaKaRa, Dalian, CHN). All the constructs were sequenced for their accuracy (Invitrogen, Beijing, CHN). The construct with “seed sequence” and matched mutated one was transfected into HEK293 with let-7b mimics or negative control respectively in 24-well plate with Lipofectamine 2000 (Invitrogen, California, US) in DMED (Gibco, California, US). After incubating (37 °C, 5% CO_2_) for 48h, the cells were lysed in RIPA (Thermo, Illinois, US). The absolute fluorescent was measured by infinite M200 multimode microplate readers (Tecan, Männedorf, CH), and it was normalized as relative fluorescent by total protein.

**Plasmid Construction and Lentivirus Packaging**

Lentivirus vector pLVT516-let-7b-sh, which silenced hsa-let-7b, and negative control (NC) vector pLVT5 were constructed by Sunbio (Shanghai, CHN). In brief, fragments containing a blocking site for let-7b (5’-ccgg***AACCACACAACCTACTACCTCA***TTTTTTG-3’ and 5’-aattCAAAAAA***TGAGGTAGTAGGTTGTGTGGTT***-3’) or stem-loop NC ones (5’-ccgg***TTCTCCGAACGTGTCACGT***TTCAAGAGA***ACGTGACACGTTCGGAGAA***TTTTTG-3’ and 5’-aattCAAAAA***TTCTCCGAACGTGTCACGT***TCTCTTGAA***ACGTGACACGTTCGGAGAA***-3’) were synthesized and annealed to form the cohesive-end inserts. The inserts were inserted into the Age I and EcoR I sites of linearized pMagic5.1/hU6/CMV/RFP vector. PCD/NL-BH*DDD and pLTR-G were co-transfected into 293T cell with pLVT516-let-7b-sh and pLVT5 respectively, and the virus particles were collected by ultracentrifugation after passing 0.45μm filter. The titer was assayed by dilution counting of red fluorescent protein (RFP), and was expressed as transducing unit (TU) / mL.

**Lentivirus Infection**

Human PAECs were seeded in 96-well plate at 5×10^3^/well 24 h before infection. The cells were infected with pLVT516-let-7b-sh or pLVT5 in serum-free ECM at multiplicity of infection (MOI) of 5, 10, 20, 30 and 40 respectively, and the culture medium was changed into ECM supplemented with FBS and ECGS after 12 h. Observing the fluorescence 24 h to 72 h after infection, and taking the minimum MOI which could reach > 90% RFP positive cells for further experiments.

**SiRNA / miRNA mimics Preparation and Transfection**

SiRNA oligonucleotides with two thymidine residues (dTdT) at the 3’-end of the sequence and let-7b mimics were purchased from GenePharma (GenePharma, Shanghai, CHN). The siRNA oligonucleotides were selected to correspond to the sequence of human TGFBR1 gene starting at the site of 351. When at 70% confluence, PAECs were transfected with siRNA oligonucleotides / let-7b mimics using Lipofectamine RNAiMAX reagent (Invitrogen, California, US) at concentration of 10 pmol per cm^2^ dish or well of plate following the manufacture. Briefly, the HPAECs were washed with serum-free medium, and cultured in it. The transfection complex (siRNA / let-7b mimics and transfection reagent mixture) was added to the medium in a drop-wise manner. Mix gently by shaking the medium back and forth. After 6 h culture at 37 °C in 5% CO_2_, the culture medium was changed into ECM supplemented with FBS and ECGS. Continuously culture for 48h, the cells and culture medium were collected for western blotting or ELISA respectively, and the wound healing assay was done at certain time point.

**Western Blotting**

The cells were harvested by chilled RIPA (Thermo, Illinois, US), and centrifuged in 12000 rpm for 15 min at 4 °C. The supernatant was collected as protein sample and 40 μL total protein was separated by sodium dodecyl sulfate-polyacrylamide gel electrophoresis (SDS-PAGE) with 10% polyacrylamide concentration and transferred onto nitrocellulose membranes (Millipore, Billerica, Massachusetts, US). After blocked in 5% defatted milk for 2 h, the membrane was incubated with primary antibody (dilution, 1:500; CST, Virginia, US) overnight. The membrane was washed in TBS buffer 3 times, and then incubated in second antibody (dilution, 1:10000; Rockland, US) for 1 h at room temperature. Following washing, the immunoblotting signal was visualized and analyzed in [Odyssey Infrared Imaging System](http://www.cnpg.com/Video/flatFiles/752/index.aspx) (LI-COR Biosciences, Rockland, US). The value of the relative density of target protein band was normalized to the density of the β-actin band. The ratio of control group was regarded as 100%.

**Enzyme-Linked Immunosorbent Assay (ELISA)**

Plasma and culture media ET-1 level was detected by Human Endothelin-1 QuantiGlo ELISA Kit (R&D System Inc, Minnesota, US), and the luminescence was detected by chemiluminescence immune detection system (YDME, Beijing, CHN). The culture media was collected from 24-well plate 48 h after transfection of siRNA for TGFBR1 and negative control respectively, and the total cell number was counted for normalization of ET-1 level. The procedure of ELISA in brief: Add 100 μL of Assay Diluent to each well, followed by 100 μL of Standard, or sample. Cover with the adhesive strip, and incubate for 1.5 h at room temperature on a horizontal orbital microplate shaker. Aspirate each well and wash for 4 times, remove any remaining Wash Buffer after last time. Add 200 μL of ET-1 Conjugate to each well, and incubate for 3 h at room temperature on the shaker. Repeat wash for 4 times. Add 100 μL of Working Glo Reagent to each well. Incubate for 5-20 min at room temperature on the benchtop protecting from light before detection.

**Wound Healing Assay**

PAECs and PASMCs were seeded in 6-well plate at an optimal density which would reach 60-70% confluence 24 h after. Scratch on the monolayer cells with a blue pipette tip perpendicularly to the plate, and further incubate them with respective medium without growth supplements. The TGF-β inducing group was treated with 10 ng/mL TGF-β1 (R&D System Inc, Minnesota, US). Images were taken at fixed time intervals of 0, 6, 12, 24 and 48 hours after scratch with microscope (Olympus, Tokyo, Japan). For normalization purposes, the wounded area was expressed as the percentage of recovery (%R) using the equation: %R= [1–(wounded area at Tt/wounded area at T0)]*100%, where Tt is the number of hours post-injury and T0 is immediately post-injury [S8].

**S-Tables**

**Table S1. Sequences of the primers used in fluorescent reporter assay.**

| **Primer** | **Sequence** | **Products (bp)** |
| --- | --- | --- |
| ET-1 3’-UTR RP (*Xba* I) | 5’-GCTCTAGACAAAAATCAAGGACAG-3’ |  |
| ET-1 3’-UTR FP (*Not* I) | 5’-ATAAGAATGCGGCCGCCAGAGAGCGTTATGT-3’ | 869 |
| TGFBR1 3’-UTR RP (*Xba* I) | 5’-GCTCTAGATGGCACTTACTGGTATAGTACAATC-3’ |  |
| TGFBR1 3’-UTR FP (*Not* I) | 5’-ATAAGAATGCGGCCGCATGTAATTCTACAGCTTTGCCTG-3’ | 682 |
| ET-1 mut1 RP | 5’-ATGAATATGAGTCAAGCACACCTATATTG-3’ |  |
| ET-1 mut1 FP | 5’-GAAACATATCCTGATTTATGGTAGTAACA-3’ |  |
| ET-1 mut2 RP | 5’-TTTCCCACATTTAATTATAGGCACCCCAAACTCTTCCCA-3’ |  |
| ET-1 mut2 FP | 5’-TACTTCTGCCAGAGTGCAATATAGGTGAGGTAGACTCATA-3’ |  |
| ET-1 mut1&2 RP | 5’-TTTCCCACATTTAATTATAGGCACCCCAAACTCTTCCCA-3’ |  |
| ET-1 mut1&2 FP | 5’-TACTTCTGCCAGAGTGCAATATAGGTGTGCTTGACTCATA-3’ |  |
| TGFBR1 mut RP | 5’-GGTCAATTGTTCAAGCACACTGAGAGGGAACAGAA-3’ |  |
| TGFBR1 mut FP | 5’-TCCCAAATTAAAACCCAGGAGCAGATCTGAAG-3’ |  |

**Table S2. Differences in clinical characteristics between microarray cohort and validation cohort.**

| **Characteristics** | **miRNA microarray** | **qRT-PCR** | ***P*-value** |
| --- | --- | --- | --- |
| **Age (yr)*** | 49.8 (10.1) | 51.9 (11.6) | 0.602 |
| **Sex (Male/Total)** | 6/10 | 25/40 | 1.000 |
| **DVT (Positive/Total)** | 6/10 | 12/30 | 0.300 |
| **MBP (mmHg)*** | 91.2 (4.4) | 93.3 (12.0) | 0.604 |
| **sPAP (mmHg)*****†** | 86.3 (13.5) | 89.5 (19.7) | 0.635 |
| **mPAP (mmHg)*****†** | 54.6 (13.3) | 51.5 (12.0) | 0.481 |
| **PVR (dyn🞄s🞄cm^-5^)*****†** | 1071.2 (243.9) | 1079.4 (598.1) | 0.914 |
| **CI (L🞄min^-1^🞄m^-2^)*****†** | 2.0 (0.6) | 2.0 (0.6) | 0.967 |
| **WHO functional class** |  |  | 0.334 |
| **I** | 1/10 | 2/40 |  |
| **II** | 2/10 | 19/40 |  |
| **III** | 6/10 | 16/40 |  |
| **IV** | 1/10 | 3/40 |  |
| **6MWD (m)*****‡** | 374.6 (27.0) | 355.5 (109.0) | 0.789 |
| **AVR (Positive/Total)** | 1/9 | 6/36 | 1.000 |
| **NTproBNP (pg/mL)*****§** | 1436.6 (1577.1) | 1456.2 (1433.5) | 0.973 |
| **CRP (mg/L)*****ǁ** | 6.0 (6.0) | 4.7 (4.2) | **0.000** |

Definition of abbreviations: DVT, deep venous thrombosis; MBP, mean blood pressure; sPAP, systolic pulmonary arterial pressure; mPAP, mean pulmonary arterial pressure; PVR, pulmonary vascular resistance; CI, cardiac index; 6MWD, 6-minute walk distance; AVR, acute vascular reaction; BNP, brain natriuretic peptide; CRP, C-reactive protein. *Mean (SD); for microarray cohort, † n=10, ‡ n=7, § n=8, ǁ n=8; and for validation (qRT-PCR) cohort, † n=36, ‡ n=31, § n=34, ǁ n=29. *P* value was calculated by Mann-Whitney *U* test, two-sample Kolmogorov-Smirnov test or $\chi^{2}$ test**.**

**Table S3. Properties of miRNAs of CTEPH patients and healthy controls differentially expressed in microarray.**

| **microRNA** | **Health controls** | |  | **CTEPH patients** | | ***P*-value** | **FDR** | **Fold**  **change** |
| --- | --- | --- | --- | --- | --- | --- | --- | --- |
|  | **Mean** | **SD** |  | **Mean** | **SD** |
| **Up-regulated** |  |  |  |  |  |  |  |  |
| **miR-1260** | 0.173 | 0.073 |  | 0.678 | 0.430 | 0.000 | 0.002 | 3.911 |
| **miR-424** | 0.009 | 0.010 |  | 0.019 | 0.018 | 0.042 | 0.047 | 2.158 |
| **miR-602** | 0.082 | 0043 |  | 0.168 | 0.120 | 0.002 | 0.007 | 2.055 |
| **miR-18b*** | 0.042 | 0.024 |  | 0.087 | 0.111 | 0.031 | 0.042 | 2.039 |
| **miR-193a-3p** | 0.007 | 0.010 |  | 0.014 | 0.018 | 0.005 | 0.012 | 1.906 |
| **miR-129-5p** | 0.544 | 0.158 |  | 1.007 | 0.802 | 0.007 | 0.016 | 1.850 |
| **miR-1908** | 4.147 | 0.965 |  | 6.670 | 3.206 | 0.002 | 0.007 | 1.608 |
| **miR-516b** | 0.013 | 0.009 |  | 0.021 | 0.009 | 0.028 | 0.040 | 1.601 |
| **miR-483-5p** | 1.654 | 0.417 |  | 2.576 | 1.458 | 0.016 | 0.027 | 1.558 |
| **miR-494** | 0.073 | 0.032 |  | 0.110 | 0.092 | 0.034 | 0.043 | 1.503 |
| **miR-933** | 0.714 | 0.140 |  | 1.044 | 0.379 | 0.005 | 0.013 | 1.462 |
| **miR-183*** | 0.281 | 0.043 |  | 0.399 | 0.116 | 0.002 | 0.007 | 1.422 |
| **miR-205** | 0.050 | 0.036 |  | 0.065 | 0.041 | 0.048 | 0.050 | 1.295 |
| **miR-585** | 0.077 | 0.024 |  | 0.087 | 0.148 | 0.023 | 0.037 | 1.128 |
| **miR-518a-3p** | 0.013 | 0.011 |  | 0.013 | 0.017 | 0.002 | 0.007 | 1.039 |
| **Down-regulated** |  |  |  |  |  |  |  |  |
| **miR-140-3p** | 0.211 | 0.130 |  | 0.024 | 0.015 | 0.018 | 0.015 | 0.107 |
| **miR-125a-3p** | 0.034 | 0.022 |  | 0.004 | 0.001 | 0.004 | 0.001 | 0.156 |
| **miR-93** | 0.332 | 0.232 |  | 0.067 | 0.075 | 0.043 | 0.075 | 0.160 |
| **miR-130a** | 0.149 | 0.079 |  | 0.047 | 0.057 | 0.023 | 0.057 | 0.182 |
| **miR-22** | 7.935 | 2.095 |  | 2.094 | 1.594 | 1.727 | 1.594 | 0.225 |
| **miR-106b** | 1.722 | 0.955 |  | 0.577 | 0.569 | 0.367 | 0.569 | 0.248 |
| **miR-222** | 0.072 | 0.034 |  | 0.020 | 0.010 | 0.017 | 0.010 | 0.274 |
| **let-7b** | 0.555 | 0.312 |  | 0.163 | 0.091 | 0.142 | 0.091 | 0.287 |
| **miR-320a** | 0.331 | 0.089 |  | 0.101 | 0.040 | 0.093 | 0.040 | 0.291 |
| **miR-425** | 0.063 | 0.028 |  | 0.020 | 0.015 | 0.016 | 0.015 | 0.306 |
| **miR-505*** | 0.064 | 0.028 |  | 0.022 | 0.013 | 0.019 | 0.013 | 0.329 |
| **miR-320b** | 0.311 | 0.057 |  | 0.115 | 0.049 | 0.106 | 0.049 | 0.347 |
| **miR-130b** | 0.077 | 0.026 |  | 0.028 | 0.012 | 0.026 | 0.012 | 0.347 |
| **miR-185** | 0.505 | 0.337 |  | 0.201 | 0.145 | 0.146 | 0.145 | 0.365 |
| **miR-30d** | 0.147 | 0.074 |  | 0.053 | 0.015 | 0.051 | 0.015 | 0.416 |
| **miR-486-5p** | 2.097 | 0.621 |  | 0.964 | 0.559 | 0.841 | 0.559 | 0.418 |
| **miR-320d** | 0.187 | 0.074 |  | 0.084 | 0.042 | 0.063 | 0.042 | 0.468 |
| **miR-320c** | 0.355 | 0.104 |  | 0.183 | 0.083 | 0.165 | 0.083 | 0.487 |
| **miR-2116** | 0.148 | 0.051 |  | 0.085 | 0.040 | 0.071 | 0.040 | 0.511 |
| **miR-423-5p** | 0.680 | 0.144 |  | 0.373 | 0.096 | 0.363 | 0.096 | 0.545 |

RVM *t*-test was applied to determine the *P-*value.

**Table S4. Diagnostic efficacy of seven methods for the 17 miRNA signature.**

| **Methods** | **Sensitivity** | **Specificity** |
| --- | --- | --- |
| **Compound Covariate Predictor** | 0.90 | 0.90 |
| **Diagonal Linear Discriminant Analysis** | 1.00 | 1.00 |
| **1-Nearest Neighbor** | 0.95 | 0.95 |
| **3-Nearest Neighbors** | 0.90 | 0.90 |
| **Nearest Centroid** | 0.95 | 0.95 |
| **Support Vector Machines** | 0.95 | 0.95 |
| **Bayesian Compound Covariate Predictor** | 0.85 | 0.85 |

**Table S5. Literature review for the functions of candidate miRNA in the signature.**

| **microRNA** | **Functions** | **Expression** |
| --- | --- | --- |
| **let-7b [S9-S11]** | Anti-oncogenic, involved in IPF | down |
| **miR-106b [S12,S13]** | Oncogenic, target genes (Smad7, p21) | down |
| **miR-140-3p [S14]** | Anti-oncogenic | down |
| **miR-185 [S15]** | Anti-oncogenic | down |
| **miR-22 [S16,S17]** | Anti-oncogenic, inhibit migartion, invasion and cell cycle | down |
| **miR-320a/b/c [S18]** | Anti-oncogenic | down |
| **miR-423-5p [S19]** | Associated with left heart insufficiency, mechanism UN | down |
| **miR-486-5p [S20]** | Anti-oncogenic | down |
| **miR-93 [S21,S22]** | Oncogenic | down |
| **miR-129-5p [S23]** | Anti-oncogenic | up |
| **miR-1908** | UN | up |
| **miR-1260** | UN | up |
| **miR-483-5p [S24,S25]** | Anti-oncogenic, inhibit angiogenesis | up |
| **miR-602 [S26]** | Oncogenic | up |
| **miR-933** | UN | up |

UN: unknown.

**Table S6. Differences of circulating miRNA levels between different conditions of clinical characteristics.**

| **Characteristics** | **miR-602** | |  | **let-7b** | |  | **miR-22** | |
| --- | --- | --- | --- | --- | --- | --- | --- | --- |
|  | ***Z*** | ***P*-value** |  | ***Z*** | ***P*-value** |  | ***Z*** | ***P*-value** |
| **Sex (Male *vs.* Female)** | -1.411 | 0.158 |  | -0.992 | 0.321 |  | -0.721 | 0.476 |
| **Post PTE (Positive *vs.* Negative)** | -0.879 | 0.379 |  | -0.068 | 0.946 |  | -1.014 | 0.310 |
| **DVT (Positive *vs.* Negative)** | -0.508 | 0.611 |  | -0.085 | 0.933 |  | -0.381 | 0.703 |
| **WHO functional class (I&II *vs.* III&IV)** | -1.449 | 0.147 |  | -1.639 | 0.101 |  | -1.259 | 0.208 |
| **AVR (Positive *vs.* Negative)** | -0.679 | 0.497 |  | **-2.037** | **0.042** |  | **-2.886** | **0.004** |
| **ACA (Positive *vs.* Negative)** | -0.801 | 0.423 |  | -1.650 | 0.099 |  | **-2.027** | **0.043** |
| **PEA (Yes *vs.* No)** | -0.899 | 0.369 |  | -1.823 | 0.068 |  | -1.720 | 0.085 |

Definition of abbreviations: PTE, pulmonary thromboembolism; DVT, deep venous thrombosis; AVR, acute vascular reaction; ACA, anticardiolipin antibody; PEA, pulmonary endarterectomy. *P* value was calculated by logistics regression analysis.

**Table S7. Top ten powerfully target pathways of let-7b by DIANA-miRPath.**

| **KEGG pathway** | **Found genes** | **-ln(*P*-value)** | **Gene name** |
| --- | --- | --- | --- |
| MAPK signaling pathway | 19 | 14.34 | MAP4K3, MAP4K4, DUSP4, ***TGFBR1***, PDGFB, MAP3K7IP2, DUSP9, FASLG, CACNA1D, DUSP16, MAP3K3, PAK1, FGF11, ACVR1B, PLA2G3, NLK, DUSP1, ACVR1C, CASP3 |
| ECM-receptor interaction | 8 | 8.89 | COL1A1, COL3A1, FNDC3A, THBS1, COL5A2, ITGB3, COL1A2, COL4A1 |
| Pancreatic cancer | 7 | 7.26 | ***TGFBR1***, RALB, RB1, ACVR1B, ACVR1C, BCL2L1, CCND1 |
| O-Glycan biosynthesis | 4 | 6.17 | GALNT2, GALNT1, GALNTL2, GCNT4 |
| Focal adhesion | 12 | 6.17 | PDGFB, COL1A1, COL3A1, VAV3, PAK1, THBS1, COL5A2, ITGB3, COL1A2, CCND2, COL4A1, CCND1 |
| Colorectal cancer | 7 | 5.66 | DVL3, ***TGFBR1***, ACVR1B, ACVR1C, FZD4, CASP3, CCND1 |
| Chronic myeloid leukemia | 6 | 4.5 | ***TGFBR1***, RB1, ACVR1B, ACVR1C, BCL2L1, CCND1 |
| TGF-beta signaling pathway | 6 | 3.37 | E2F5, ***TGFBR1***, GDF6, THBS1, ACVR1B, ACVR1C |
| Glycan structures-biosynthesis 1 | 7 | 3.09 | B3GNT1, NDST2, GALNT2, GALNT1, GALNTL2, MGAT4A, GCNT4 |
| Jak-STAT signaling pathway | 8 | 2.8 | SOCS4, IL13, OSMR, GHR, IL10, CCND2, BCL2L1, CCND1 |

The potential target genes of let-7b were predicted by intersection of Targetscan and PicTar.

**References**

S1. Barst RJ, McGoon M, Torbicki A, Sitbon O, Krowka MJ, et al. (2004) Diagnosis and differential assessment of pulmonary arterial hypertension. J Am Coll Cardiol 43: 40S-47S.

S2. Man TK, Chintagumpala M, Visvanathan J et al. Expression profiles of osteosarcoma that can predict response to chemotherapy. Cancer research 2005;65:8142-50.

S3. Dudoit S, Fridlyand J, Speed TP (2002) Comparison of discrimination methods for the classification of tumors using gene expression data. Journal of the American Statistical Association: 77-87.

S4. Lewis DP, Jebara T, Noble WS (2006) Support vector machine learning from heterogeneous data: an empirical analysis using protein sequence and structure. Bioinformatics 22: 2753-2760.

S5. Radmacher MD, McShane LM, Simon R (2002) A paradigm for class prediction using gene expression profiles. J Comput Biol 9: 505-511.

S6. Ashburner M, Ball CA, Blake JA, Botstein D, Butler H, et al. (2000) Gene ontology: tool for the unification of biology. The Gene Ontology Consortium. Nat Genet 25: 25-29.

S7. Kanehisa M, Goto S, Kawashima S, Okuno Y, Hattori M (2004) The KEGG resource for deciphering the genome. Nucleic Acids Res 32: D277-280.

S8. Lauder H, Frost EE, Hiley CR, Fan TP (1998) Quantification of the repair process involved in the repair of a cell monolayer using an in vitro model of mechanical injury. Angiogenesis 2: 67-80.

S9. Ohshima K, Inoue K, Fujiwara A, Hatakeyama K, Kanto K, et al. (2010) Let-7 microRNA family is selectively secreted into the extracellular environment via exosomes in a metastatic gastric cancer cell line. PLoS One 5: e13247.

S10. Pandit KV, Corcoran D, Yousef H, Yarlagadda M, Tzouvelekis A, et al. (2010) Inhibition and role of let-7d in idiopathic pulmonary fibrosis. Am J Respir Crit Care Med 182: 220-229.

S11. Schultz J, Lorenz P, Gross G, Ibrahim S, Kunz M (2008) MicroRNA let-7b targets important cell cycle molecules in malignant melanoma cells and interferes with anchorage-independent growth. Cell Res 18: 549-557.

S12. Ivanovska I, Ball AS, Diaz RL, Magnus JF, Kibukawa M, et al. (2008) MicroRNAs in the miR-106b family regulate p21/CDKN1A and promote cell cycle progression. Mol Cell Biol 28: 2167-2174.

S13. Smith AL, Iwanaga R, Drasin DJ, Micalizzi DS, Vartuli RL, et al. (2012) The miR-106b-25 cluster targets Smad7, activates TGF-beta signaling, and induces EMT and tumor initiating cell characteristics downstream of Six1 in human breast cancer. Oncogene.

S14. Miles GD, Seiler M, Rodriguez L, Rajagopal G, Bhanot G (2012) Identifying microRNA/mRNA dysregulations in ovarian cancer. BMC Res Notes 5: 164.

S15. Liu M, Lang N, Chen X, Tang Q, Liu S, et al. (2010) miR-185 targets RhoA and Cdc42 expression and inhibits the proliferation potential of human colorectal cells. Cancer Lett 301: 151-160.

S16. Li J, Liang S, Yu H, Zhang J, Ma D, et al. (2010) An inhibitory effect of miR-22 on cell migration and invasion in ovarian cancer. Gynecol Oncol 119: 543-548.

S17. Xu D, Takeshita F, Hino Y, Fukunaga S, Kudo Y, et al. (2011) miR-22 represses cancer progression by inducing cellular senescence. J Cell Biol 193: 409-424.

S18. Schaar DG, Medina DJ, Moore DF, Strair RK, Ting Y (2009) miR-320 targets transferrin receptor 1 (CD71) and inhibits cell proliferation. Exp Hematol 37: 245-255.

S19. Tijsen AJ, Creemers EE, Moerland PD, de Windt LJ, van der Wal AC, et al. (2010) MiR423-5p as a circulating biomarker for heart failure. Circ Res 106: 1035-1039.

S20. Shen J, Liu Z, Todd NW, Zhang H, Liao J, et al. (2011) Diagnosis of lung cancer in individuals with solitary pulmonary nodules by plasma microRNA biomarkers. BMC Cancer 11: 374.

S21. Fang L, Deng Z, Shatseva T, Yang J, Peng C, et al. (2011) MicroRNA miR-93 promotes tumor growth and angiogenesis by targeting integrin-beta8. Oncogene 30: 806-821.

S22. Xu D, He XX, Chang Y, Sun SZ, Xu CR, et al. (2012) Downregulation of MiR-93 Expression Reduces Cell Proliferation and Clonogenicity of HepG2 Cells. Hepatogastroenterology 59.

S23. Brest P, Lassalle S, Hofman V, Bordone O, Gavric Tanga V, et al. (2011) MiR-129-5p is required for histone deacetylase inhibitor-induced cell death in thyroid cancer cells. Endocr Relat Cancer 18: 711-719.

S24. Qiao Y, Ma N, Wang X, Hui Y, Li F, et al. (2011) MiR-483-5p controls angiogenesis in vitro and targets serum response factor. FEBS Lett 585: 3095-3100.

S25. Wang L, Shi M, Hou S, Ding B, Liu L, et al. (2012) MiR-483-5p suppresses the proliferation of glioma cells via directly targeting ERK1. FEBS Lett 586: 1312-1317.

S26. Yang L, Ma Z, Wang D, Zhao W, Chen L, et al. (2010) MicroRNA-602 regulating tumor suppressive gene RASSF1A is overexpressed in hepatitis B virus-infected liver and hepatocellular carcinoma. Cancer Biol Ther 9: 803-808.
